# Biosynthetic investigation of γ-lactones in *Sextonia rubra* wood using *in situ* TOF-SIMS MS/MS imaging to localize and characterize biosynthetic intermediates

**DOI:** 10.1038/s41598-018-37577-5

**Published:** 2019-02-13

**Authors:** Tingting Fu, Emeline Houël, Nadine Amusant, David Touboul, Grégory Genta-Jouve, Serge Della-Negra, Gregory L. Fisher, Alain Brunelle, Christophe Duplais

**Affiliations:** 1 Institut de Chimie des Substances Naturelles, CNRS UPR 2301, Université Paris-Sud, Université Paris-Saclay, Avenue de la Terrasse, 91198 Gif-sur-Yvette, France; 2 Institut de Physique Nucléaire, UMR8608, IN2P3-CNRS, Université Paris-Sud, Université Paris-Saclay, 91406 Orsay, France; 3 CNRS UMR8172 EcoFoG, AgroParisTech, CIRAD, INRA, Université des Antilles, Université de Guyane, 97300 Cayenne, France; 4 CIRAD UMR93 EcoFoG, AgroParisTech, CNRS, INRA, Université des Antilles, Université de Guyane, 97310 Kourou, France; 5 Université Paris Descartes, UMR CNRS 8638 COMETE, 4 avenue de l’observatoire, 75006 Paris, France; 6 Physical Electronics, Chanhassen, Minnesota 55317 USA

## Abstract

Molecular analysis by parallel tandem mass spectrometry (MS/MS) imaging contributes to the *in situ* characterization of biosynthetic intermediates which is crucial for deciphering the metabolic pathways in living organisms. We report the first use of TOF-SIMS MS/MS imaging for the cellular localization and characterization of biosynthetic intermediates of bioactive γ-lactones rubrynolide and rubrenolide in the Amazonian tree *Sextonia rubra* (Lauraceae). Five γ-lactones, including previously reported rubrynolide and rubrenolide, were isolated using a conventional approach and their structural characterization and localization at a lateral resolution of ~400 nm was later achieved using TOF-SIMS MS/MS imaging analysis. 2D/3D MS imaging at subcellular level reveals that putative biosynthetic γ-lactones intermediates are localized in the same cell types (ray parenchyma cells and oil cells) as rubrynolide and rubrenolide. Consequently, a revised metabolic pathway of rubrynolide was proposed, which involves the reaction between 2-hydroxysuccinic acid and 3-oxotetradecanoic acid, contrary to previous studies suggesting a single polyketide precursor. Our results provide insights into plant metabolite production in wood tissues and, overall, demonstrate that combining high spatial resolution TOF-SIMS imaging and MS/MS structural characterization offers new opportunities for studying molecular and cellular biochemistry in plants.

## Introduction

The chemistry of natural products has always relied on new technological advances in analytical chemistry to analyze the metabolome of living organisms and to determine the chemical structure of secondary metabolites of interest in ecology, pharmacology, materials science, and agriculture. On the other hand, studying biosynthetic pathways remains a critical point for a better understanding of the diverse natural ways of molecular entities synthesis. Understanding metabolic pathways is the key step to identify enzymes that can be used in biochemistry for alternative route in the production of compounds of interest when extraction or organic synthesis fail to provide sufficient quantities for commercial purposes. In this regard many biosynthetic pathways of natural products used as drugs remain unknown and yet to be discovered. During the last decade, new developments in genomics^1^ and bioengineering^2^ have been implemented to decipher plant metabolism and these approaches are now advantageously combined with data in plant metabolomics^3^. However, methods in analytical chemistry are lacking for the *in vivo* and *in situ* detection of low-abundance intermediates in cellular compartments, which could speed up the discovery of organismal metabolic pathways. For instance, gas chromatography (GC) and liquid chromatography (LC) coupled to mass spectrometry (MS), together with nuclear magnetic resonance (NMR) spectroscopy, are the most effective methods to analyze and identify plant natural products^4–6^. However, solvent extraction of whole tissue results in the loss of spatial information in the metabolite localization, which is essential to understand the metabolism and the biological functions of specialized cells^7^. Since mass spectrometry imaging (MSI) has been well established for visualizing chemical distributions in biological samples, several studies have employed MSI to reveal the localization of plant metabolites^8,9^. Particularly, time-of-flight secondary ion mass spectrometry (TOF-SIMS) is now a routine chemical imaging technique with subcellular resolution^10^ that has been applied to map metabolite distribution in various wood species^11–13^. Furthermore, direct three-dimensional (3D) TOF-SIMS imaging can be realized by the dual beam depth profiling method in which a sputter ion beam is used to ablate the samples layer by layer, consequently revealing the in-depth distribution of chemical components^14^. TOF-SIMS prototypes providing MS/MS capability have also been developed recently^15–19^ and this technique has not been expending yet in plant metabolic studies.

To address the challenging topic of identifying *in situ* biosynthetic intermediates through TOF-SIMS tandem MS imaging, we decided to study wood metabolites in the abundant Amazonian tree species *Sextonia rubra* (Mez) van der Werff (Lauraceae), initially identified as *Nectandra rubra* (Mez) C.K. Allen and *Ocotea rubra* Mez^20^. *S*. *rubra* is a neotropical shade-tolerant rainforest tree species native to South America and is one of the most commercially exploited wood for construction in French Guiana owing to its exceptional natural durability. In 1971, two γ-lactones, rubrynolide and rubrenolide, were isolated from the stem wood of *S*. *rubra*^21–23^, of which fungicidal and termicidal activities have been reported later, suggesting that these wood metabolites are likely responsible for *S*. *rubra* heartwood natural durability^24,25^. Although the total synthesis of rubrynolide and rubrenolide has led to stereochemical revision of the stereogenic centers^26–29^, the biosynthesis of these bio-sourced γ-lactones has not been discussed since the original report^30^.

More recently we have reported the bioactivities of *S*. *rubra* heartwood extract on *Aedes aegypti* larvae for applications in vector control strategies^31^ and therefore we decided to reinvestigate *S*. *rubra* natural products. Previously we used TOF-SIMS and the novel TOF-SIMS tandem MS imaging technique to localize and characterize the two γ-lactones, rubrynolide and rubrenolide in *S*. *rubra* heartwood samples^32^. Herein we combined traditional phytochemical analysis, extraction-purification process followed by LC-MS and NMR analyses, with the novel TOF-SIMS tandem MS imaging technique to study the γ-lactone metabolic diversity in different plant organs. This original approach allows us to re-investigate the rubrynolide and rubrenolide biosynthesis in *S*. *rubra*. This strategy benefits from high mass-accuracy spectral data of isolated compounds, which facilitates metabolites identification in wood cells in TOF-SIMS imaging. *In situ* MS/MS analysis, in conjunction with LC-MS/MS analysis, provides also structural information that contributes to characterizing putative biosynthetic precursors which are present in low concentration and are not amenable to phytochemical isolation. We successfully determined the structure of five isolated γ-lactones including two original compounds rubrynolide and rubrenolide by NMR, LC-MS analysis and *in situ* TOF-SIMS tandem MS imaging. All identified γ-lactones are presumably related to rubrynolide and rubrenolide metabolic pathway. Overall, the spatial 2D/3D distribution of bioactive rubrynolide, rubrenolide and their biosynthetic precursors is discussed herein, at cellular and subcellular level in *S*. *rubra* wood samples, and in regard to the proposed revised biosynthetic route.

## Results

### Phytochemical study of Sextonia rubra tree species

**Figure 1:**
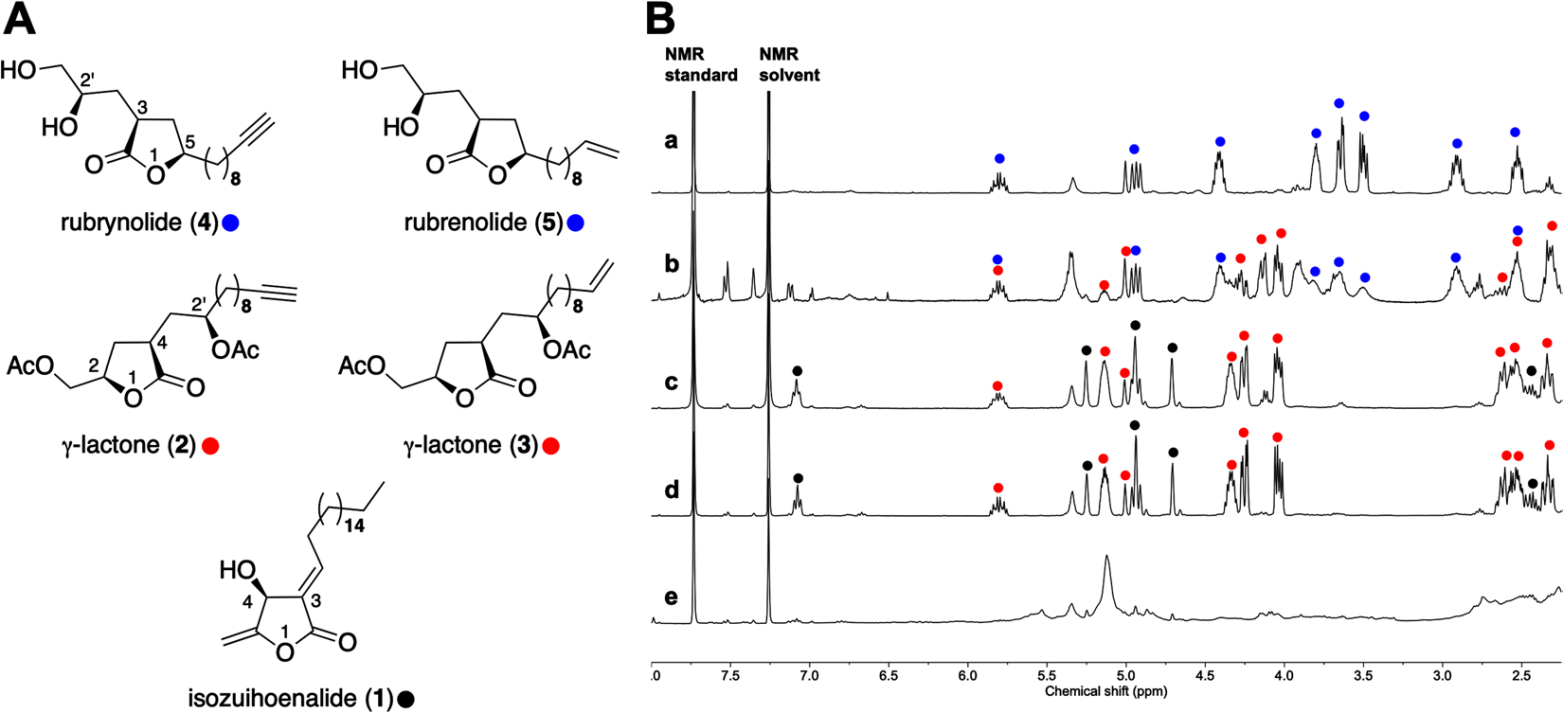
Structures of γ-lactones isolated from *Sextonia rubra* (**A**) and ^1^H qNMR zoom spectra (**B**) of heartwood (a), bark (b), sapwood (c), roots (d), and leaf (e) crude extracts. Black, red and blue dots on NMR spectra show cumulative proton signals of γ-lactones **1**, **2–3** and **4–5** respectively.

In the course of understanding the heartwood formation process of the *S*. *rubra* tree species, and along with the previous isolation of rubrynolide and rubrenolide, we conducted the phytochemical study of two *S*. *rubra* individuals (Sr1 and Sr2). While performing LC-MS analysis of ethyl acetate extracts of roots, sapwood, heartwood, bark, and leaf of Sr1 individual, we noticed that most extracts are composed of a few major constituents (Supplementary Fig. S1). Three γ-lactones **1**, **2** and **3** were consecutively isolated in Sr1 sapwood along with heartwood compounds rubrynolide (**4**) and rubrenolide (**5**), and their structural determinations were performed (Fig. 1A). Isozuihoenalide (**1**) shows a molecular formula of C_23_H_40_O_3_ determined by electrospray ionization (ESI) MS ([M + H]^+^, *m/z* 365.3051, Δ_*m/z*_ = 0.3 ppm) (Supplementary Fig. S2). ^1^H and ^13^C NMR spectra of **1** were similar to those of zuihoenalide^33^, indicating the same *β*-hydroxy-*γ*-methylene-*α*,*β*′-unsaturated-*γ*-lactone skeleton (Supplementary Table S1, Figs S3 and S4). However, contrary to the *Z* double-bond of zuihoenalide, the *E* geometry of the trisubstituted double bond was confirmed by comparing the values of ^1^H NMR chemical shifts and coupling constants of the olefinic *β*-proton [δ 7.09 ppm (1 H, td, *J* = 7.8, 1.8 Hz, H-1′)] with related butanolides isoobtusilactone A and lincomolide D^34–36^. The presence of a broad singlet δ 1.26 ppm (28 H, br s, H-4′-17′) was attributed to protons in the long methylene chain. The exocyclic olefinic protons appeared at δ 4.96 ppm, 4.72 ppm (each 1 H, d, *J* = 1.5 Hz, H_*Z*_-1″, H_*E*_-1″) and one hydroxymethine proton is located at δ 5.26 ppm (1 H, br s, H-4). The structure of isozuihoenalide was elucidated as (3*E*)-4*S*-hydroxy-5-methylene-3-octadecylidenedihydrofuran-2-one, which was further confirmed by gradient correlation spectroscopy (gCOSY), adiabatic gradient heteronuclear single-quantum correlation spectroscopy (gHSQCAD) and gradient heteronuclear multiple-bond correlation spectroscopy (gHMBC) experiments (Figures S5–S7). The absolute configuration at C-4 was established by comparison between theoretical and experimental electronic circular dichroism (ECD) spectra (Supplementary Fig. S8).

The γ-Lactones **2** and **3**, isolated in sapwood, have a molecular formula of C_21_H_32_O_6_ (MS (ESI), *m/z* 381.2281 [M + H]^+^ Δ_*m/z*_ = 2.5 ppm, *m/z* 403.2099 [M + Na]^+^ Δ_*m/z*_ = 2.0 ppm, *m/z* 783.4280 [2 M + Na]^+^ Δ_*m/z*_ = −1.3 ppm) and C_21_H_34_O_6_ (MS (ESI), *m/z* 383.2415 [M + H]^+^ Δ_*m/z*_ = −3.9 ppm, *m/z* 405.2225 [M + Na]^+^ Δ_*m/z*_ = − 5.6 ppm, *m/z* 787.4594 [2 M + Na]^+^ Δ_*m/z*_ = − 1.1 ppm) (Figs S9 and S10), respectively. ^1^H and ^13^C NMR spectra of γ-lactones **2** and **3** were similar to that of the γ-lactone previously isolated in a marine actinobacteria^37^ (Supplementary Table S2, Figs S11, S12, S16 and S17). ^1^H chemical shifts and multiplicity, in combination with the gHSQCAD spectra, indicates the presence of two carbinols (C-1 at 65.1 ppm and C-2” at 69.2 ppm) with one (C-1) in α position of an asymmetric carbon (C-2 at 79.2 ppm) bearing an oxygen atom in γ-lactone ring (Supplementary Figs S14 and S19). The lactone is substituted at C-4 (37.9 ppm) position with an aliphatic chain containing either a terminal triple bond or a terminal double bond at C-11 position in γ-lactones **2** and **3** respectively. Both carbinol oxygen atoms are acetylated as indicated by the double CH_3_ singlet signal at 2.06 and 2.08 ppm, and the double ^13^C signals at 170.7 ppm. Thus, the structure of γ-lactones **2** and **3** were elucidated as (4-(2-acetoxydodec-11-yn-1-yl)-5-oxotetrahydrofuran-2-yl)-methyl acetate and (4-(2-acetoxydodec-11-en-1-yl)-5-oxotetrahydrofuran-2-yl)-methyl acetate respectively, which was further confirmed by gCOSY, gHSQCAD and gHMBC experiments (Supplementary Figs S13–S15 and S18–S20). Moreover, the γ-lactones **2** and **3** have similar specific rotation values (Supplementary Table S2) and the (2 *R*, 4 *R*, 2′*R*) stereochemistry of γ-lactones **2** was established by comparison between the theoretical and experimental ECD spectra (Supplementary Fig. S21).

The ^1^H NMR spectra of ethyl acetate extracts exhibits resolution that is suitable for integrating characteristic proton signals of γ-lactones **1**–**5**. The quantification of metabolites in different plant organs of the *S*. *rubra* Sr1 individual was assessed by quantitative NMR spectroscopy (Supplementary Table S3, Figs S22–S26). The aligned ^1^H NMR spectra in Fig. 1B highlight the presence of γ-lactones in the roots, sapwood and heartwood in relatively high purity for a crude plant extract. The results support those found previously in the LC-MS analysis of the Sr1 individual (Supplementary Fig. S1) except for rubrynolide **4** and rubrenolide **5** that were detected in sapwood. A possible explanation is that it can be difficult to accurately distinguish between sapwood and transition zone tissues and the LC-MS spectra of Sr1 sapwood extract may be contaminated with the transition zone tissue. The leaf extract is a complex mixture of metabolites with no major compound whereas the ^1^H NMR spectrum of the heartwood extract shows exclusively the presence of rubrynolide **4** and rubrenolide **5** proton signals. The bark extract has indeterminate quantity of rubrynolide **4** and rubrenolide **5** along with little quantity of γ-lactones **2** and **3** and other unknown metabolites. Roots and sapwood have similar extraction yields of isozuihoenalide **1** (2.1% and 1.9% respectively, Table S3) as well as combined γ-lactones **2** and **3** (6% and 5.3% respectively). Interestingly rubrynolide **4** and rubrenolide **5** are present in high concentration in *S*. *rubra* heartwood and represent 8.1% by weight of the acetyl acetate extract in a 2:1 ratio.

### *In situ* identification of γ-lactones

**Figure 2:**
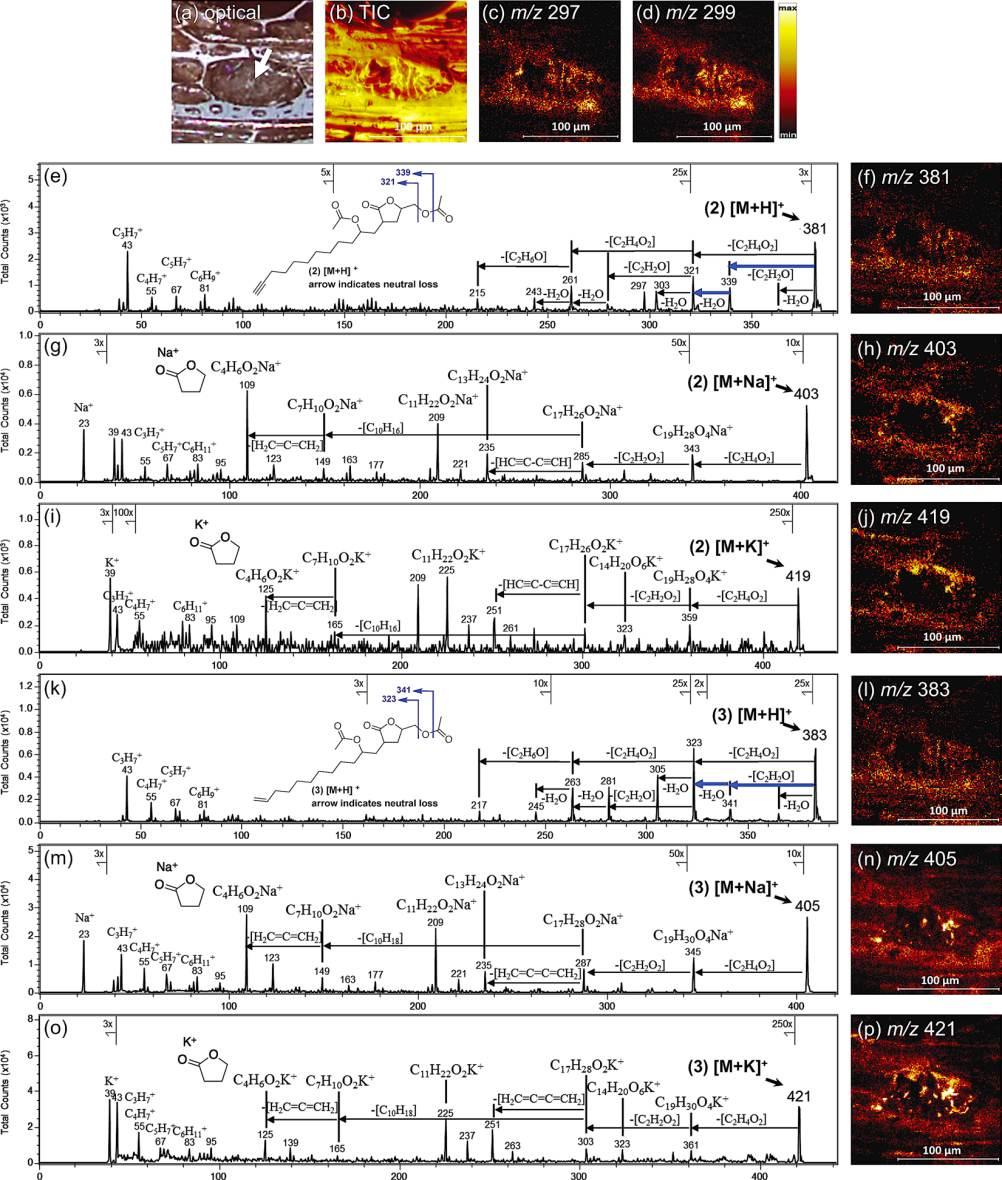
TOF-SIMS tandem MS imaging and product ion peak attributions. (**a**) Optical image of sapwood surface. (**b**) MS^1^ total ion (TIC) image. MS^1^ images of *m/z* 297 ions γ-lactone **4** rubrynolide (**c**) and *m/z* 299 ions γ-lactone **5** rubrenolide (**d**) were shown to give a comparison with the cellular localization of γ-lactones **2** and **3**. (**f**) MS^2^ TIC image of *m/z* 381 precursor ion. The product ion attributions shown in panel (**e**), MS/MS spectrum of the [M + H]^+^ precursor ion at *m/z* 381, support the structure of γ-lactone **2**. (h) MS^2^ TIC image of *m/z* 403 precursor ion. The product ion attributions shown in panel (**g**), MS/MS spectrum of the [M + Na]^+^ precursor ion at *m/z* 403, support the structure of γ-lactone **2**. (**j**) MS^2^ TIC image of *m/z* 419 precursor ion. The product ion attributions shown in panel (**i**), MS/MS spectrum of [M + K]^+^ precursor ion at *m/z* 419, support the structure of γ-lactone **2**. (**l**) MS^2^ TIC image of *m/z* 383 precursor ion. The product ion attributions shown in panel (**k**), MS/MS spectrum of the [M + H]^+^ precursor ion at *m/z* 383, support the structure of γ-lactone **3**. (**n**) MS^2^ TIC image of *m/z* 405 precursor ion. The product ion attributions shown in panel (**m**), MS/MS spectrum of the [M + Na]^+^ precursor ion at *m/z* 405, support the structure of γ-lactone **3**. (**p**) MS^2^ TIC image of *m/z* 421 precursor ion. The product ion attributions shown in panel (**o**), MS/MS spectrum of [M + K]^+^ precursor ion at *m/z* 421, support the structure of γ-lactone **3**. Each ion image has a field-of-view of 150 μm x 150 μm with a pixel dimension of 586 nm. In panels **e** and **k** the characteristic neutral losses are indicated, while in panels **g**–**j**, **m**–**p** the alkali metal adducts of oxolan-2-one are shown at *m/z* 109 and *m/z* 125.

For the herein study we use the TOF-SIMS tandem MS imaging technique that we previously applied for *in situ* identification of rubrynolide **4** and rubrenolide **5** in *S*. *rubra* transition zone wood^32^. γ-lactones **2** and **3** were further identified on the sapwood surface of *S*. *rubra* Sr2 individual without any pretreatment (Fig. 2). Tandem MS imaging analyses were performed on a 150 µm × 150 µm analytical wood area where an oil cell was present (Fig. 2a). TOF-SIMS MS/MS spectra and imaging of precursors at *m/z* 381.22 [M + H]^+^ (γ-lactone **2**) and *m/z* 383.24 [M + H]^+^ (γ-lactone **3**) are illustrated in Fig. 2e and k, f and l, respectively. The low mass fragments display regular occurrence of 12 amu or 14 amu mass intervals, which is typical of long hydrocarbon chain fragmentation. While the high mass range spectra show the protonated precursor ions as well as the characteristic fragments originated from the loss of acetate groups: *e*.*g*. −42 amu (-CH_2_=C=O) and −60 amu (-CH_3_CO_2_H). The *in situ* MS/MS data is consistent with extraction followed by LC-MS analysis where the peak annotation is achieved within 5 ppm mass accuracy (Supplementary Figs S9 and S10). Ion images of precursors at *m/z* 381 and *m/z* 383 were obtained by collecting all the fragment ions and the remaining precursors after the CID collisional activation.

The γ-lactones **2** and **3** are co-localized in the oil cell within the analyzed sapwood area (Fig. 2f,l) with their corresponding alkalized (Na^+^ and K^+^) adducts (Fig. 2h,j, 2n,p) which further confirmed the structure and lateral distribution of these two γ-lactones. Loss of [C_10_H_16_] and [C_10_H_18_] fragments (carbon chain) are characteristic in rubrynolide and rubrenolide MS^2^ spectra and are also observed in MS^2^ spectra of alkalized γ-lactone **2** and **3** (Fig. 2g–j,m–p). The C_4_H_6_O_2_K^+^ and C_4_H_6_O_2_Na^+^ ions (γ-butyrolactone ions) appear for each alkalized adducts at strong signal intensity. All γ-lactones ions including rubrynolide **4** and rubrenolide **5** were detected in the same cellular environment (Fig. 2c–p).

### The presence of non-isolated γ-lactones

Considering the number of carbon atoms in the structures of γ-lactones **2** and **3** (C_17_ without acetyl moieties), the corresponding biosynthetic precursors are unlikely to originate from isozuihoenalide **1** (C_23_) but presumably from a C_17_ analog of isozuihoenalide **1**. Therefore, we searched for chemical entities with a molecular formula of C_17_H_28_O_3_. By further mining the LC-MS data of *S*. *rubra* wood extracts, we successfully extracted the mass spectra of two possible biosynthetic intermediates which can be assigned as γ-lactone **6** and γ-lactone **8** in the sapwood extract. γ-Lactone **6** is a C_17_ analog of isozuihoenalide **1** with a terminal double bond in the hydrocarbon chain (Supplementary Fig. S27). Different adduct forms [M + H]^+^ (*m/z* 279.1961, Δ_*m/z*_ = 0.4 ppm, [M + Na]^+^ (*m/z* 301.1779, Δ_*m/z*_ = −0.3 ppm) and [2 M + Na]^+^ (*m/z* 579.3666, Δ_*m/z*_ = 0.7 ppm) are all present in the MS^1^ spectrum. In the MS^2^ spectrum of the protonated γ-lactone **6**, the low mass range ions are typical fragments from hydrocarbon chain and the high mass range fragments are produced via neutral losses of H_2_O and CO from C_17_H_27_O_3_^+^, which correspond well to the structure shown in Supplementary Fig. S27. γ-Lactone **8** is an acetylated product of γ-lactone **6** (Supplementary Fig. S28) and same fragment ions are observed in both MS^2^ spectra in the mass range below *m/z* 279.1961. TOF-SIMS tandem MS imaging was then carried out for *in situ* characterization of these newly found γ-lactones in sapwood. On the analyzed area in Supplementary Fig. 29, four ions at *m/z* 279, *m/z* 281, *m/z* 321, and *m/z* 323 were detected, of which *m/z* 279 and *m/z* 321 are assigned as γ-lactone **6** and **8** respectively as in LC-MS analysis and *m/z* 281 and *m/z* 323 are γ-lactone **7** and **9**, respectively. The MS^2^ spectra of precursor ions at *m/z* 279 and *m/z* 281 show interference peaks apart from the fragment ions derived from protonated γ-lactones **6** and **7**. Whereas fragmentation of precursor ions at *m/z* 321 and *m/z* 323 give well resolved MS^2^ spectra and produce characteristic fragments of *m/z* 279 and *m/z* 281 respectively through the losses of H_2_O and CH_2_CO, indicating the presence of acetyl function group. Therefore, although the absolute structures of γ-lactones **6**–**9** cannot be determined by NMR characterization due to the low abundance in the wood samples, their presence in the sapwood is shown in both TOF-SIMS and LC-MS analyses.

### 2D and 3D localization of γ-lactones

**Figure 3:**
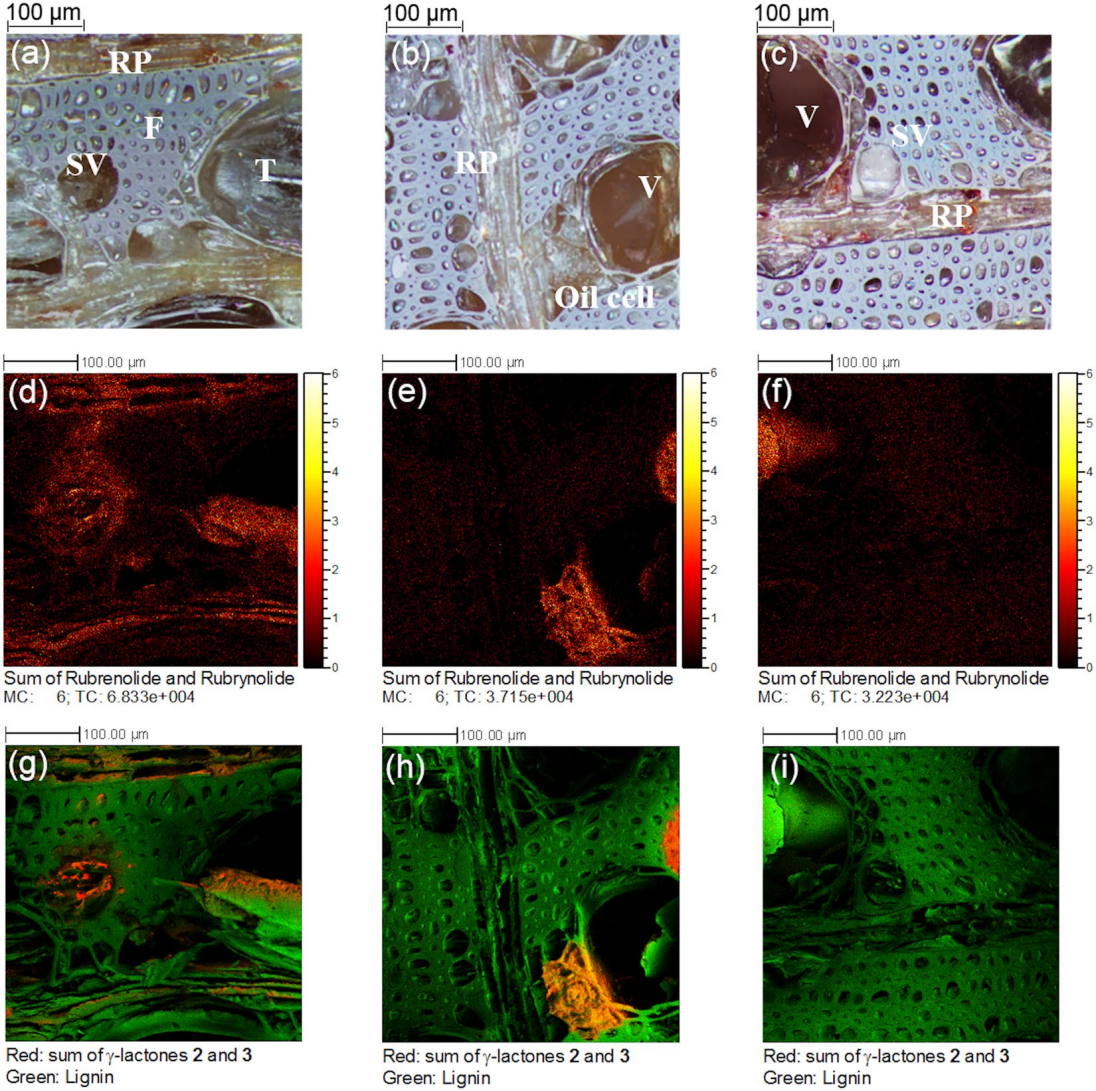
Distribution of secondary metabolites from sapwood to heartwood. (**a**–**c**): Optical images of sapwood (**a**), transition zone (**b**), and heartwood (**c**). RP: Ray parenchyma cell; F: Fiber cell; SV: small vessel; V: Vessel; T: Tyloses. (**d**–**f**): Ion images of rubrynolide (γ-lactone **4**, *m/z* 297 [M + H]^+^) and rubrenolide (γ-lactone **5**, *m/z* 299 [M + H]^+^) in sapwood (**d**), transition zone (**e**), and heartwood (**f**). (**g**–**i**): Two-color overlay ion images of γ-lactones **2** and **3** and lignin fragments in sapwood (**g**), transition zone (**h**) and heartwood (**i**). Ion images summed for γ-lactones **2** and **3** are: γ-lactone **2**, *m/z* 381 [M + H]^+^, *m/z* 403 [M + Na]^+^, *m/z* 419 [M + K]^+^; γ-lactone **3**, *m/z* 383 [M + H]^+^, *m/z* 405 [M + Na]^+^, *m/z* 421, [M + K]^+^. Lignin fragments^63^ used were: C_8_H_9_O_2_^+^ (*m/z* 137.06), C_9_H_11_O_2_^+^ (*m/z* 151.07), C_8_H_8_O_3_^+^ (*m/z* 152.05), C_8_H_9_O_3_^+^ (*m/z* 153.06), C_9_H_9_O_3_^+^ (*m/z* 165.06), C_9_H_11_O_3_^+^ (*m/z* 167.07) and C_10_H_13_O_3_^+^ (*m/z* 181.08), C_11_H_9_O_3_^+^ (*m/z* 189.06).

It has been demonstrated that heartwood is formed from sapwood after the death of parenchyma cells^38^. Therefore, we mapped by TOF-SIMS, with high lateral resolution, the surface from sapwood to heartwood to investigate the localization of wood metabolites before and after heartwood formation. The optical and ion images displayed in Fig. 3 were recorded on analytical areas of 400 µm × 400 µm of sapwood, transition zone, and heartwood, respectively. Not surprisingly, the summed protonated ion images of rubrynolide **4** (*m/z* 297) and rubrenolide **5** (*m/z* 299) show identical distribution in the wood tissues (Supplementary Fig. S30). For the considered wood surfaces, they are observed in ray parenchyma cells and tyloses (which are expansions of parenchyma cells in the lumen of vessels) within sapwood, oil cells in transition zone, and tyloses in heartwood (Fig. 3d,f). The presence of rubrynolide **4** and rubrenolide **5** in the sapwood is probably due to the fact that the sapwood is sampled very close to the transition zone where they are biosynthesized and abundant. It should be noted that the protonated rubrynolide and rubrenolide were detected at lower intensity in heartwood, despite being largely abundant as revealed by LC-MS and NMR analyses, which is likely due to the low ionization efficiency in this wood tissue due to a possible matrix effect. After confirming the same cellular localization of individual compounds (Supplementary Fig. S30), ion images of γ-lactones **2**–**3** are summed and highlighted (red) in the two-color overlay with the ion images of lignin fragments (green). It is revealed that γ-lactones **2** and **3**, are co-localized in the same cell types as rubrynolide (**4**) and rubrenolide (**5**) except that they are barely present in the heartwood which confirms NMR and LC-MS analysis (Fig. 3d–i).

**Figure 4:**
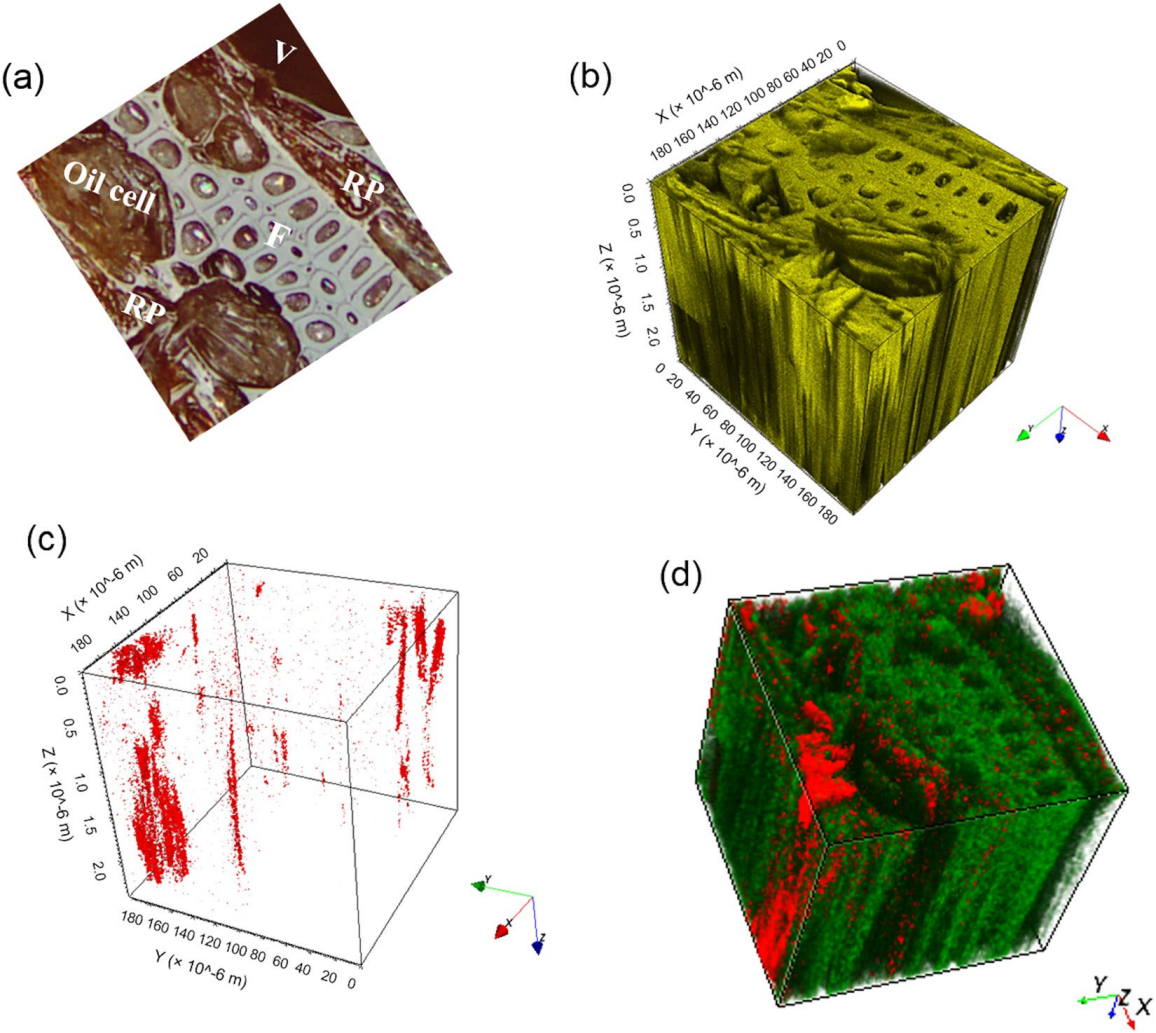
3D distribution of γ-lactone **3**. (**a**) Optical image of the wood sample from transition zone. (**b**) 3D reconstruction of total ions. (**c**) 3D reconstruction of the *m/z* 421 ion ([M + K]^+^, γ-lactone **3**). (**d**) Two-color overlay of *m/z* 421 ion ([M + K]^+^, γ-lactone **3**) and lignin fragment^63^ C_4_H_3_^+^. Red: *m/z* 421; Green: C_4_H_3_^+^. The sputter depth and sputter dose are 2.42 µm and 1.38 × 10^16^ ions/cm^2^, respectively.

In order to track the in-depth distribution of wood metabolites, three-dimensional analyses were carried out on the transition zone wood sample using dual beam depth profiling and imaging. A 200 µm × 200 µm area comprising various cell types was chosen for the analysis (Fig. 4a) and the resulting total ion volume is depicted in Fig. 4b. Owing to the large number of constituents in the argon clusters, the impact energy of individual atoms is extremely low, leading to minimum damage of the samples. Therefore, wood structure and different wood cells are readily recognizable even after a sputter dose of 1.38 × 10^16^ ions/cm^2^ (Supplementary Fig. S31), and the metabolites diffusion or degradation can be negligible. The sputter depth was calculated at 2.42 µm by calibrating the erosion rate with a 20 µm thick *S*. *rubra* wood section. The 3D reconstruction of [M + K]^+^ ion of γ-lactone **3** at *m/z* 421 (Fig. 4c), as well as its overlay with lignin fragment ion C_4_H_3_^+^ (Fig. 4d), illustrates the metabolic distribution in ray parenchyma and oil cells, matching the lateral distribution.

## Discussion

The Lauraceae family includes 2850 known plant species in about 45 genera and has a wide distribution in tropical and subtropical regions. Numerous studies have reported the past and present use of Lauraceae plants by human populations for various purposes, including construction^39^, drugs^40^, spice^41^ and perfume^41^. The chemical diversity of natural products in Lauraceae plants is relatively diverse and includes alkaloids, arylpropanoids, nitro-derivatives, 2-pyrones, flavonoids, benzophenones, terpenoids, fatty acids and γ-lactones^42^. Several obtusilactone analogs like isozuihoenalide **1**, have been characterized in the genus *Lindera*^43^, *Machilus*^44^ and *Cinnamomum*^45^, and have attracted attention due to their *in vitro* and *in vivo* cytotoxic activities against HeLa cells and several cancer cell lines^36,46–48^. As mentioned previously, rubrynolide **4** and rubrenolide **5**, originally isolated in *S*. *rubra* wood^21^ and recently in *Mezilaurus* genus^49^, have fungicide and termicidal properties that support the ecological role of wood protection against pathogens and have economic value as bio-insecticides. In the current report, our results highlight for the first time the presence, in the same plant species, of these structurally related γ-lactones **1**–**5**, consequently, bring up the question of the biosynthetic relationship between these γ-lactones in Lauraceae.

**Figure 5:**
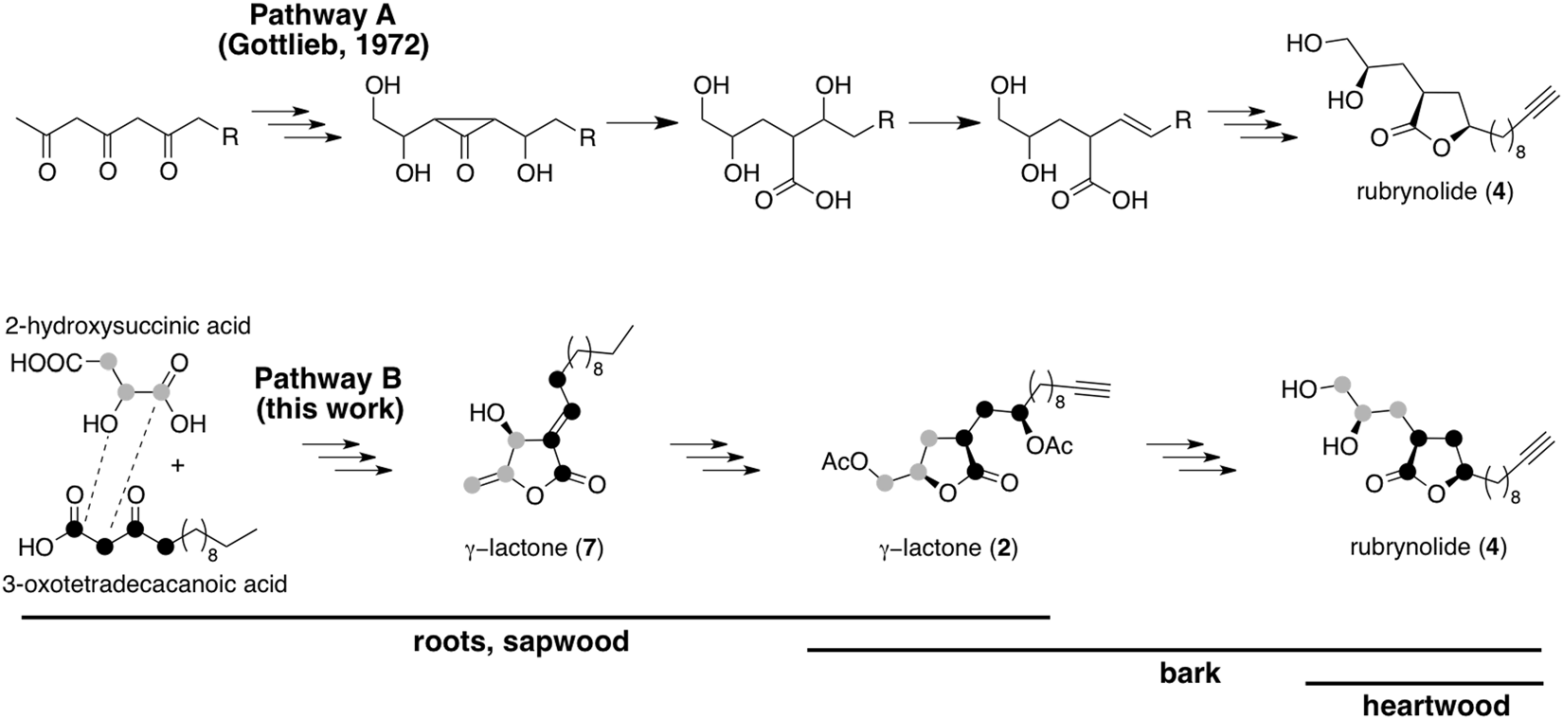
Putative formation of rubrynolide (**4**). Biosynthesis pathways proposed by Gottlieb (pathway A) and in this work (pathway B).

The biosynthesis of rubrynolide in *S*. *rubra*, originally proposed by Gottlieb, starts with the Favorsky-type rearrangement of a polyketide precursor to form a cyclo-propanone (Fig. 5). The suggested mechanism explains how one single carbonyl is excluded from the polyketide chain in the final γ-lactone wood products^30^. However, our results provide new insights.

First, NMR spectroscopy demonstrates the presence of isozuihoenalide **1** and γ-lactones **2**–**3** in *S*. *rubra* roots, sapwood, and transition zone whereas rubrynolide **4** and rubrenolide **5** are found in heartwood. TOF-SIMS tandem MS imaging analysis provides *in situ* characterization of γ-lactones **2**–**3**, **4**–**5** and evidence of the presence of some non-isolated γ-lactones in sapwood. Additionally, high-resolution multi-area TOF-SIMS imaging shows the spatial distribution of the relevant metabolites across the whole wood surface from sapwood to heartwood. The γ-lactones (**1**–**5**) are localized in some specific cell types (ray parenchyma cells, oil cells, tyloses-occluded vessels) suggesting the occurrence of the biosynthesis of these compounds in the same cellular compartments, in agreement with our previous results on rubrynolide (**4**) and rubrenolide (**5**) colocalization^32^. Considering that tyloses in heartwood arise from ray parenchyma cells before expanding into the adjacent vessel through cell wall pits^50^ and knowing that oil cells in Lauraceae are enlarged or vertical ray parenchyma cells^51^, the biosynthesis of γ-lactones **1**–**5** presumably occurs in ray parenchyma cells in agreement with previous observations^12,52,53^.

Secondly, we hypothesize that γ-lactones **1**–**5** biosynthesis is part of the same metabolic pathway, and we propose an alternative biogenic route to rubrynolide which starts with the reaction between 2-hydroxysuccinic acid and 3-oxotetradecanoic acid (Fig. 5). In this pathway, an esterification reaction is followed by an aldol-type ring closure between the *β*-keto ester enol and carboxyl group of the succinic acid to form γ-lactone **7**. Although precursors 2-hydroxysuccinic acid and 3-oxotetradecanoic acid were not detected in current analysis, the γ-lactone ring formation is very likely to be similar to the biosynthesis of carolic and carlosic acids in *Penicillium charlesii* demonstrated by ^14^C isotopic enrichment^54^ and closely related to the formation of several γ-lactone natural products^55,56^. Consecutive reduction, oxidation, and acetylation steps produce γ-lactone **2** as a single (2 *R*, 4 *R*, 2′*R*) stereoisomer from γ-lactone **7**. Lastly, following a deacetylation reaction, the transesterification of γ-lactone **2** secondary free alcohol occurs in a stereoselective manner to give rubrynolide **4** (2 *R*, 4 *R*, 2′*R*) stereoisomer. The same biochemical process gives γ-lactone **3** and rubrenolide **5**. This new pathway is further supported by the detection of the non-isolated intermediates γ-lactones **6**–**9** in the sapwood sample.

Deacetylated analogs of γ-lactones **2**, **3** were not detected in quantitative amount in regard to the concentration of γ-lactones **4**, **5** in the transition zone, which suggests that deacetylation and transesterification reactions likely occur in concert, thus preventing the accumulation of deacetylated analogs in wood cells. Surprisingly, the corresponding rubrynolide analogs of γ-lactone **1** are not present in *S*. *rubra* transition zone or heartwood which raises the question of γ-lactone **1** outcome during the heartwood formation. Overall, TOF-SIMS tandem MS imaging is a very powerful tool for *in situ* structural characterization as demonstrated in the case of γ-lactones **2**, **3**. Although further investigations using either ^13^C-isotopic enrichment experiments or ^13^C NMR analysis at natural abundance^57^ will likely provide unambiguous evidence to support or to disprove this pathway, we demonstrate herein that TOF-SIMS tandem MS imaging is a promising and complementary analytical method for tracking biosynthetic intermediates in biological samples.

## Methods

### Plant material

The samples were taken from two *Sextonia rubra* adult trees (Sr1 and Sr2) from the Paracou forest in French Guiana (5°15′ N, 52°55′W) in December 2015. A wood increment core of each individual was taken at height of about 1~1.5 m and stored in freeze condition (−18 °C) until imaging analysis. The trees were chosen as representative of the species (adult, without damage and with an intact crown) according to the method used by Rutishauser *et al*.^58^.

### Extraction and isolation

Extractions of sapwood and heartwood from *S*. *rubra* Sr1 individual were performed according to previously described procedures^32^. Briefly, 200 g of wood material ground into small particle of 0.5 mm were extracted three times at room temperature for 48 h with ethyl acetate (3 × 500 mL). The ethyl acetate extracts were then purified by column chromatography (EtOAc). Preparative chromatography was performed at 15 mL/min with a W600 pump and a W2487 double wavelength UV detector (Waters) using a Discovery C18 column (15 cm × 21.2 mm, 5 µm, Supelco). HPLC analyses were performed on a Discovery C18 column (15 cm × 4.6 mm, 5 µm, Supelco) at 1 mL/min using a Waters HPLC system equipped with a W2996 photodiode array absorbance detector and a W2424 light-scattering detector. Water (HPLC grade) was obtained from a Milli-Q system (Milli-Q plus, Millipore Bedford, MA).

Isolation of isozuihoenalide **1** was carried out on 3 mL of a sapwood extract solution at a concentration of 50 mg/mL in water/acetonitrile 40:60 using a linear gradient of water/acetonitrile (40:60 to 0:100 over 20 min) and remaining at 100% acetonitrile during 10 min. The eluates from the column were monitored at 210 and 240 nm wavelengths. Fractions containing the same constituents according to analytic chromatographic profiles obtained at 210 and 240 nm and with ELSD detection were gathered and evaporation allowed the isolation of compound **1** (22 mg) in pure form. Isolation of butanolides **2** and **3** was carried out on 3 mL of a sapwood extract solution at a concentration of 50 mg/mL in water/acetonitrile 40:60 using the same procedure. Compounds **2** (43.4 mg, 28.9% w/w) and **3** (18.1 mg, 12.1% w/w) were both isolated in pure form.

### LC-MS analysis

Purified products from *S*. *rubra* Sr1 were prepared at a concentration of 0.1 mg/mL in methanol (Sigma-Aldrich, Saint Quentin-Fallavier, France). LC-MS/MS experiments were performed on a HPLC Ultimate 3000 system (Dionex, Voisins-le-Bretonneux, France) coupled with an Agilent 6540 Q-ToF (Agilent Technologies, Waldbronn, Germany) tandem mass spectrometer. LC separation was achieved with an Accucore RP-MS column (100 × 2.1 mm, 2.6 µm, Thermo Scientific, Les Ulis, France) with a mobile phase consisting of water with 0.1% formic acid (A) and acetonitrile with 0.1% formic acid (B). Compounds were eluted at a flow rate of 0.4 mL/min with a gradient from 5% B to 100% B in 25 min and then 100% B for 3 min. Injection volume was fixed at 5 µL for all the analyses. Mass spectra were recorded with an electrospray ion source in positive ion mode with the following parameters: spray voltage set at 3.5 kV, capillary temperature at 325 °C, capillary voltage at 45 V and fragmentor voltage at 120 V. The collision energy was optimized and fixed at 15 eV for all the MS/MS fragmentation acquisitions except that 30 eV was chosen for alkalized ions. Internal calibration was achieved with two calibrants (*m/z* 121.0509 and *m/z* 922.0098) providing a high mass accuracy of approximately 2 ppm. Mass resolution (FWHM, full width at half maximum) is 20,000 at *m/z* 922 in MS and MS/MS spectra.

### NMR analysis

^1^H NMR spectra were recorded at 400 MHz and ^13^C NMR spectra at 100.6 MHz on a Varian 400 NMR spectrometer equipped with a 5 mm inverse probe (Auto X PGF 1 H/15N-13C). Samples were dissolved in deuterated chloroform (CDCl_3_) in 5 mm tubes as stated. Chemical shifts are in ppm downfield from tetramethylsilane (TMS), and coupling constants (J) are in Hz (s stands for singlet, d for doublet, t for triplet, q for quartet, m for multiplet, br for broad). NMR analyses were all performed on *S*. *rubra* Sr1 individual. For the structural determination of each isolated compound, two 5 mm NMR tubes containing 8 mg and 25 mg of pure compound dissolved in deuterated chloroform (CDCl_3_) were prepared for 1 H and 2D sequences respectively. All sequences ^1^H (64 scans), ^13^C (2560 scans), gCOSY (4 scans × 256 increments), gHSQCAD (4 scans × 512 increments), gHMBC (4 scans × 512 increments) were recorded with an acquisition time of 1.0 s, a relaxation delay of 1.0 s for a 45° pulse sequence. All NMR spectra were phased and baseline corrected with MestReNova 9.0 (Mestrelab Research) before being processed.

Quantitative NMR analyses were performed as follows. Wood extracts (14 mg) and the standard for quantitative NMR (TraceCERT^®^) 1,2,4,5-tetrachloro-3-nitrobenzene (CAS: 117-18-0, Sigma-Aldrich ref.^7^, 40384 mg) were dissolved in deuterated chloroform (CDCl_3_, 400 µL) in a 2 mL vial before transferring the solution into a 5 mm NMR tube. NMR acquisition time was set at 2.55 s with a spectral width of 6410.3 Hz and 16 scans were recorded with a relaxation delay of 5 × T1 (70 s) for a 45° pulse sequence. ^1^H spectra were processed with MestReNova 9.0 (Mestrelab Research) software to perform phase and baseline corrections. For quantification of isozuihoenalide **1**, ^1^H signals corresponding to protons (7.09, 5.26 and 4.72 ppm) were manually integrated and the mean value of the integrations was used to calculate the concentration of isozuihoenalide **1** in samples. For quantification of γ-lactones **2** and **3**, ^1^H signals corresponding to protons (4.33, 4.24 and 4.02 ppm) were manually integrated, the mean value of the integrations and the mean molecular mass (M = 381.22 g.mol^−1^) were used to calculate the concentration of γ-lactones **2** and **3** in samples. For quantification of rubrynolide **4** and rubrenolide **5**, ^1^H signals corresponding to protons (3.80, 3.64 and 3.49 ppm) were manually integrated, the mean value of the integrations and the mean molecular mass (M = 297.22 g.mol^−1^) were used to calculate the concentration of rubrynolide **4** and rubrenolide **5** in samples.

### Determination of the absolute configuration of isozuihoenalide 1 and γ-lactone 2

All density functional theory (DFT) calculation have been performed using Gaussian 16^59^. A conformational analysis was performed using the GMMX module using the MMFF94 force field and a 3.5 kcal/mol energy window. Due to the high flexibility of both compounds, no major conformers were identified and the ECD calculations were performed on the most stable conformer after geometry optimization. The B3LYP method at the 6–31 g(d) level was used for all DFT calculations (OPT, FREQ and TD). The time dependent (TD) calculations were realized for 20 excited states. The SpecDis 1.71 software was used to plot the ECD spectra^60^. The experimental ECD spectra were acquired on a Jobin–Yvon model C8 spectropolarimeter calibrated with (1 *S*)-(+)-10-camphorsulfonic acid.

### Ultramicrotoming of wood samples for TOF-SIMS imaging

The wood increment core of *S*. *rubra* Sr2 individual was cut by an electric saw into small wood blocks (~0.7 cm × 0.7 cm × 0.7 cm) corresponding to sapwood, transition zone and heartwood, respectively. Each block was then trimmed with a razor blade to generate a transverse cutting surface of approximately 1 mm × 2.5 mm which was left to be cut with an ultramicrotome (EM UC6, Leica Microsystèmes, SAS, Nanterre, France) using a diamond knife (DIATOME Cryotrim 45°, Leica Microsystèmes, SAS, Nanterre, France). A high cutting speed of 50 mm/s was used considering the dense transverse wood surface and the cutting feed was set at 200 nm. The clearance angle was kept constant at 6° during the sectioning. Optical images of the wood surfaces were acquired at 10×magnification with an Olympus BX51 microscope (Rungis, France) equipped with a motorized scanning stage (Marzhauser Wetzlar GmbH, Wetzlar, Germany) and a SC30 color camera, and monitored by the Olympus Stream Motion 1.9 software. Extended focal imaging (EFI) scanning mode was used to reveal the topography of the wood surface.

### TOF-SIMS analysis

Mass spectrometry imaging acquisitions were performed with a commercial TOF-SIMS IV (ION-TOF GmbH, Münster, Germany) mass spectrometer equipped with a bismuth liquid metal ion gun and an argon cluster ion source. Mass spectra and ion density images were recorded using Bi_3_^+^ cluster ions as the primary ion beam with a kinetic energy of 25 keV. In order to simultaneously obtain high spatial and high mass resolution on wood surface bearing a diversity of topographic attributes, the so-called burst alignment ion focusing mode was applied with a delayed extraction of secondary ions as previously described^61^. The primary ion pulse duration was set at 100 ns and the current measured at 10 kHz was 0.07 pA. After the extraction delay, secondary ions were extracted and first accelerated to a kinetic energy of 2 keV and then post-accelerated to 10 keV before reaching a hybrid detector composed of a single microchannel plate followed by a scintillator and a photomultiplier. A low energy pulsed electron flood gun (20 eV) was applied to neutralize the charges accumulated on the insulating wood surface. 2D imaging acquisitions were performed on areas of 400 μm × 400 μm with 1024 × 1024 pixels, thus a pixel size of 400 nm × 400 nm, and a primary ion dose density of 3 × 10^12^ ions/cm^2^. 3D imaging was achieved with the dual beam depth profiling method with 25 keV Bi_3_^+^ as analysis beam and 10 keV Ar_1000_^+^ as sputter beam, respectively. Depth profile in positive polarity was acquired by alternatively analyzing 200 μm × 200 μm area (divided by 512 × 512 pixels, thus ~0.4 μm/pixel) and sputtering 500 μm × 500 μm area. The sputter depth was calibrated by a 20 µm thick transverse wood section of *S*. *rubra* (Supplementary Fig. S32). A sputter depth of 2.42 μm was then calculated with a sputter dose of 1.38 × 10^12^ ions/cm^2^. For the 3D imaging experiment. Data processing was performed using SurfaceLab 6.5 (ION-TOF GmbH, Münster, Germany), and the primary ion beam shift during the acquisitions were corrected by ‘lateral shift correction’ function in the software. Due to the extraction delay, the relationship between time-of-flight and the square root of *m/z* can no longer be considered linear as otherwise generally agreed in mass calibration of secondary ions with low initial kinetic energy distribution^62^. Internal mass calibration was therefore achieved by taking advantage of the lignin and polysaccharide fragments derived from the wood samples^13,63^.

### *In situ* TOF-SIMS tandem MS imaging analysis

*In situ* MS/MS identification was performed using a PHI *nanoTOF* II TOF-SIMS Parallel Imaging MS/MS instrument (Physical Electronics, Minnesota, U.S.A.)^18^. This instrument has a mass resolution of 10000 and 3000 for the MS and MS/MS mass spectra respectively and the mass accuracy is lower than 5 ppm. In this spectrometer, a precursor selector allows the deflection of a desired proportion of precursor ions within a monoisotopic selection window into a collision cell at ~1.5 keV where argon is used as collision gas. The resulting product ions as well as the remaining precursors are then mass resolved and detected within a linear TOF analyzer. This instrumental design enables simultaneous recording of MS (MS^1^) and MS/MS (MS^2^) images comprised of a full mass spectrum at each image pixel. The ion images and spectra were collected over an area of 150 µm × 150 µm, with 256 × 256 pixels and an ion dose of ≤2 × 10^13^ ions/cm^2^, using a primary ion beam of Bi_3_^+^. To avoid charge accumulation on the insulating surface, low energy electrons (≤25 eV) and low energy Ar^+^ ions (≤10 eV) were employed for charge compensation. Mass spectra were acquired over a mass range of *m/z* 0−1000 in positive ion mode and mass calibration was achieved with internal fragments. Data processing was performed using PHI TOF-DR (Physical Electronics, Minnesota, U.S.A.) software^18^.

## Supplementary information

SI revised
