# Supplementary material for: Biosynthetic investigation of γ-lactones in Sextonia rubra wood using in situ TOF-SIMS MS/MS imaging to localize and characterize biosynthetic intermediates
Source: Sci Rep. 2019 Feb 13;9:1928. doi: 10.1038/s41598-018-37577-5 (PMC6374367; doi:10.1038/s41598-018-37577-5)

Supplementary information

Biosynthetic investigation of γ-lactones in *Sextonia rubra* wood using *in situ* TOF-SIMS MS/MS imaging to localize and characterize biosynthetic intermediates

Tingting Fu[1,2], Emeline Houël[3], Nadine Amusant[4], David Touboul[1], Grégory Genta-Jouve[5], Serge Della-Negra[2], Gregory L. Fisher[6,*], Alain Brunelle[1], Christophe Duplais[3,*]

[1]Institut de Chimie des Substances Naturelles, CNRS UPR 2301, Université Paris-Sud, Université Paris-Saclay, 91198 Gif-sur-Yvette, France
[2]Institut de Physique Nucléaire, UMR8608, IN2P3-CNRS, Université Paris-Sud, Université Paris-Saclay, 91406 Orsay, France
[3]CNRS, UMR EcoFoG, AgroParisTech, CIRAD, INRA, Université des Antilles, Université de Guyane, 97300 Cayenne, France
[4]CIRAD, UMR EcoFoG, AgroParisTech, CNRS, INRA, Université des Antilles, Université de Guyane, 97310 Kourou, France
[5]Université Paris Descartes, UMR CNRS 8638 COMETE, 4 avenue de l'observatoire, 75006 Paris, France.
[6]Physical Electronics, Chanhassen, Minnesota, 55317 USA

Figure S1: Total Ion Current (TIC) chromatogram of ethyl acetate extracts of leaves, bark, heartwood, sapwood and roots of S. rubra (individual Sr1) 4
Figure S2: MS1 and MS2 spectra of isozuihoenalide 1 from roots extract 5
Table S1: NMR data of isozuihoenalide 1 6
Figure S3: 1H NMR spectrum of isozuihoenalide 1 7
Figure S4: 13C NMR spectrum of isozuihoenalide 1 8
Figure S5: gCOSY NMR spectrum of isozuihoenalide 1 9
Figure S6: gHSQCAD NMR spectrum of isozuihoenalide 1 10
Figure S7: gHMBC NMR spectrum of isozuihoenalide 1 11
Figure S8: Experimental and predicted ECD spectra of isozuihoenalide 1 12
Figure S9: MS1 and MS2 spectra of γ-lactone 2 from sapwood extract 13
Figure S10: MS1 and MS2 spectra of γ-lactone 3 from sapwood extract 14
Table S2: NMR data of γ-lactones 2 and 3 15
Figure S11: 1H NMR spectrum of γ-lactone 2 17
Figure S12: 13C NMR spectrum of γ-lactone 2 18
Figure S13: gCOSY NMR spectrum of γ-lactone 2 19
Figure S14: gHSQCAD NMR spectrum of γ-lactone 2 20
Figure S15: gHMBC NMR spectrum of γ-lactone 2 21
Figure S16: 1H NMR spectrum of γ-lactone 3 22
Figure S17: 13C NMR spectrum of γ-lactone 3 23
Figure S18: gCOSY NMR spectrum of γ-lactone 3 24
Figure S19: gHSQCAD NMR spectrum of γ-lactone 3 25
Figure S20: gHMBC NMR spectrum of γ-lactone 3 26
Figure S21: Experimental and predicted ECD spectra of γ-lactone 2 27
Table S3: Extraction yields of γ-lactones from roots, sapwood, bark, and heartwood extracts by 1H NMR quantification 28
Figure S22: 1H NMR spectra of bark extracts 29
Figure S23: 1H NMR spectra of sapwood extracts 30
Figure S24: 1H NMR spectra of heartwood extracts 31

Figure S25: 1H NMR spectra of root extracts 32

Figure S26: 1H NMR spectra of leaf extracts 33

Figure S27: MS1 and MS2 spectra of protonated ion at m/z 279.1961 from sapwood extract 34

Figure S28: MS1 and MS2 spectra of protonated ion at m/z 321.2064 from sapwood extract 35

Figure S29: TOF-SIMS tandem MS imaging and product ion peak attributions 36

Figure S30: Distribution of γ-lactones 2-3, γ-lactone 4 rubrynolide, γ-lactone 5 rubrenolide, in sapwood (SW), transition zone (TZ) and heartwood (HW), respectively. 38

Figure S31: Total ion images of the analytical area at different depth during the argon cluster sputtering 39

Figure S32: Argon cluster sputter depth measurement 40

Figure S1: Total Ion Current (TIC) chromatogram of ethyl acetate extracts of leaves, bark, heartwood, sapwood and roots of S. rubra (individual Sr1). The main metabolites (**1**-**5**) are indicated in (d) and (f).

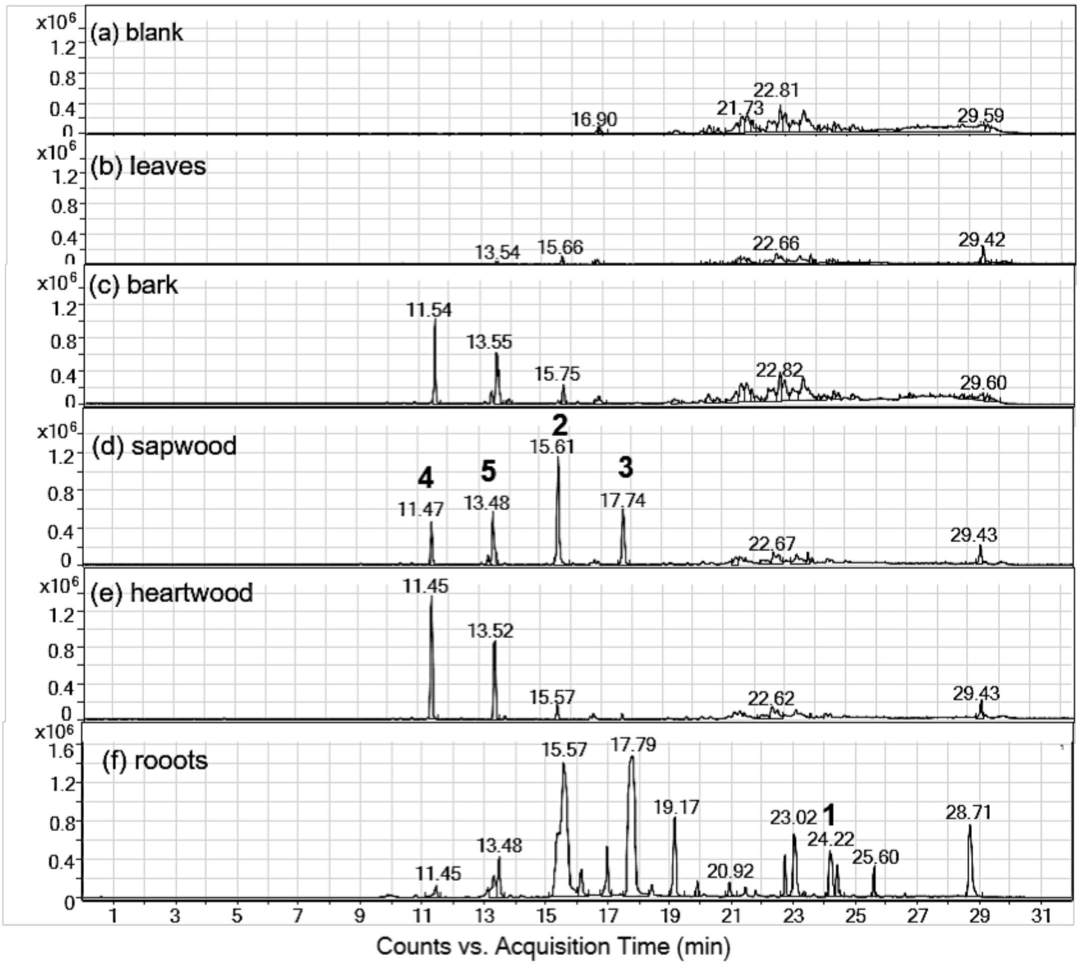

Note that the presence of γ-lactones **4** and **5** and the absence of γ-lactones **1** in sapwood (d) is not representative of sapwood LC-MS profile. This indicates that the sample (d) was contaminated with transition zone tissue as the discrimination between the sapwood and transition zone can be difficult.

Figure S2: MS1 and MS2 spectra of isozuihoenalide 1 from roots extract (RT= 24.22 min, Supplementary Fig S1-f)

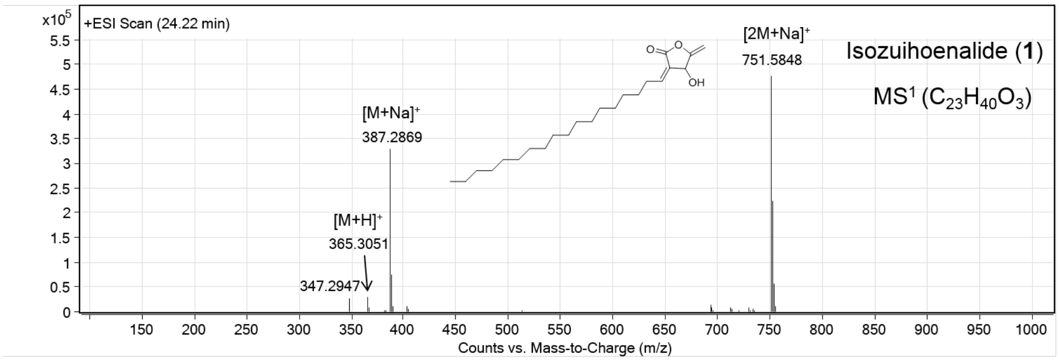

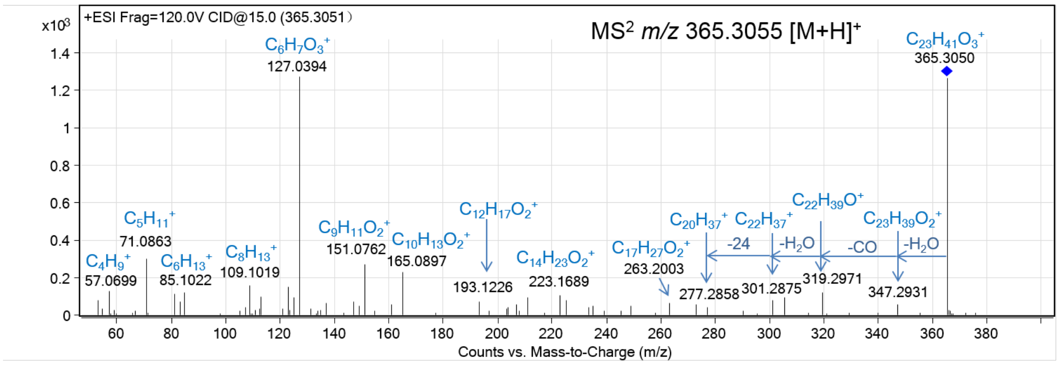

Table S1: NMR data of isozuihoenalide 1.

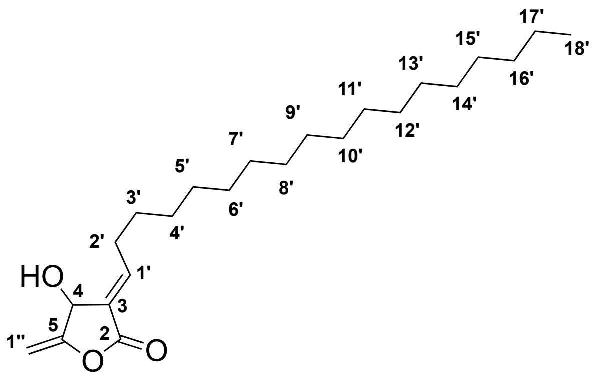

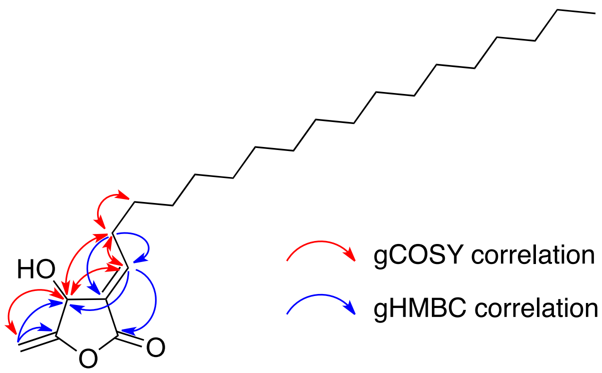

N°	^{13}C (δ)	^{1}H (δ)	gCOSY (^{1}H-^{1}H)	gHMBC (^{1}H-^{13}C)
2	166.68 (C)			
3	127.44 (C)			
4	66.66 (CH)	5.26 (1H, br s)	H1’-H2’-H1”	
5	157.83 (C)			
1’	150.35 (CH)	7.09 (1H, td, J = 7.8, 1.8 Hz)	H2’-H4	C2 - C4
2’	29.8-28.4 (CH2)	2.46 (2H, m)	H1’-H1”-H3’-H4	C1” - C3
3’	29.8-28.4 (CH2)	1.53 (2H, t, J = 7.2 Hz)	H2’	
4’-15’	29.8-28.4 (CH2)	1.26 (28H, br s)		
16’	32.0 (CH2)	1.26 (28H, br s)		
17’	22.8 (CH2)	1.26 (28H, br s)		
18’	14.2 (CH2)	0.88 (3H, t, J = 6.9 Hz)		
1”	91,48 (CH2)	4.96 (1H, d, J = 1.5 Hz, H_Z-1”)<br>4.72 (1H, d, J = 1.5 Hz, H_E-1”)	H4 - H2’	C4 - C5

Figure S3: 1H NMR spectrum of isozuihoenalide 1.

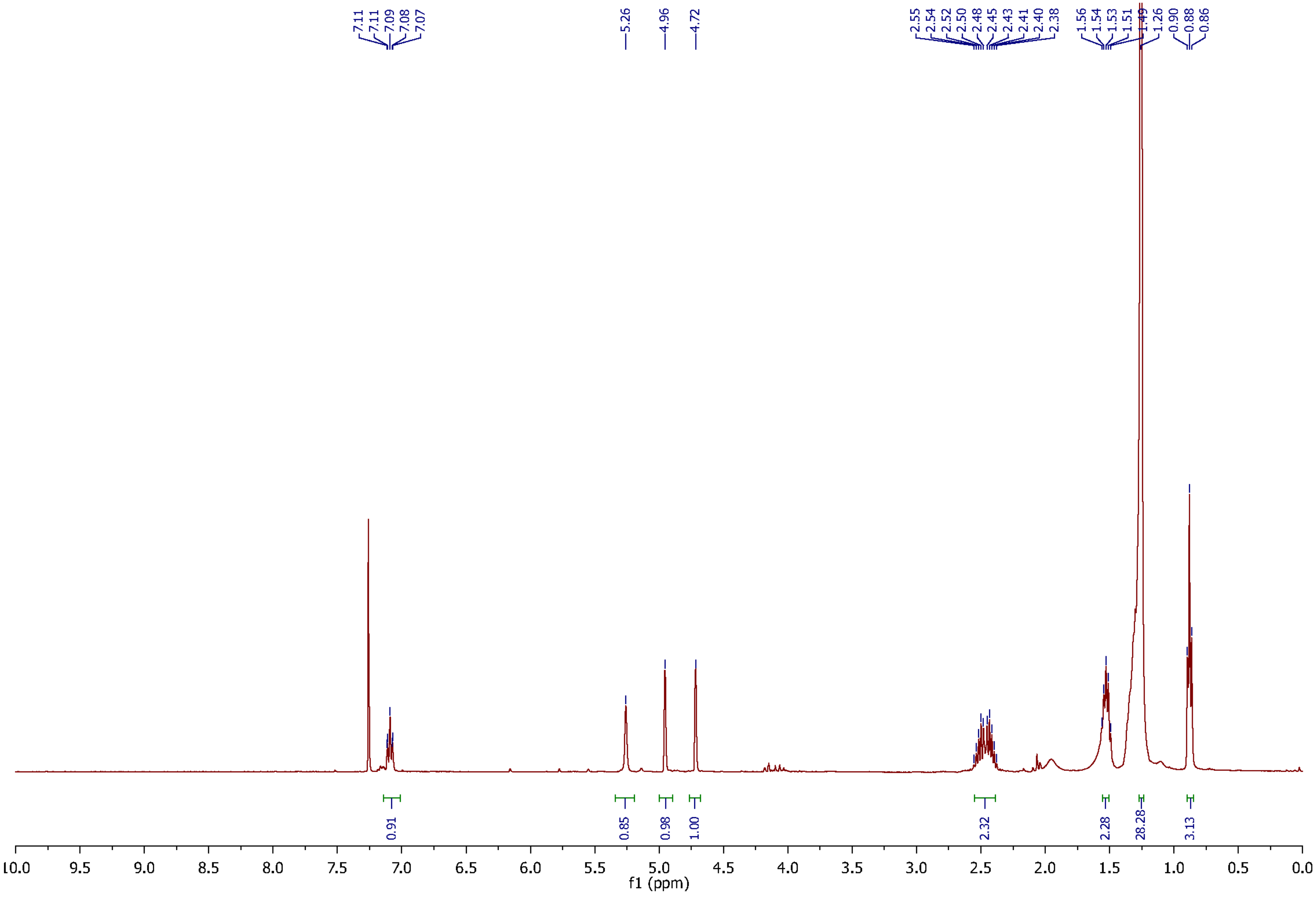

Figure S4: 13C NMR spectrum of isozuihoenalide 1.

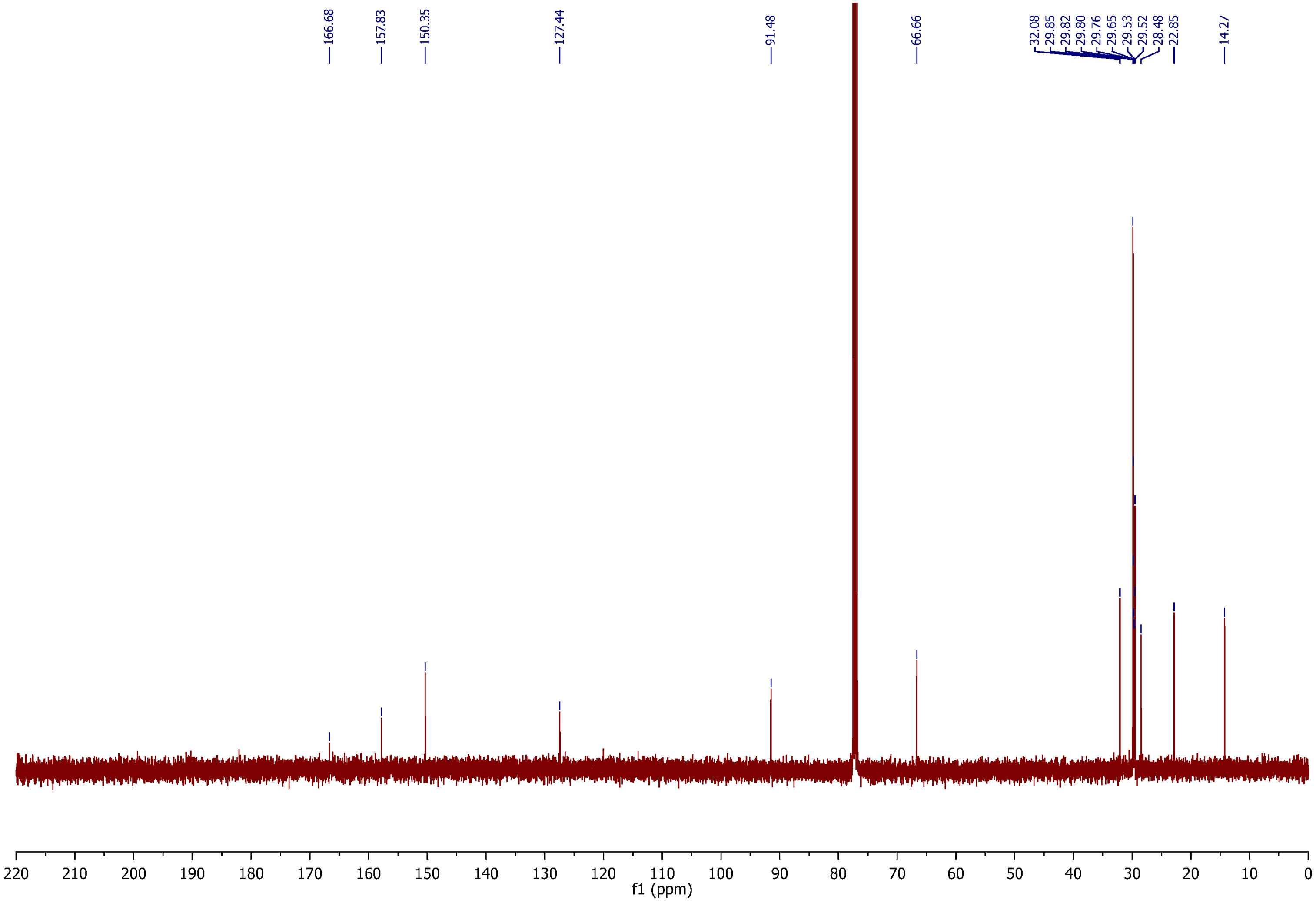

Figure S5: gCOSY NMR spectrum of isozuihoenalide 1.

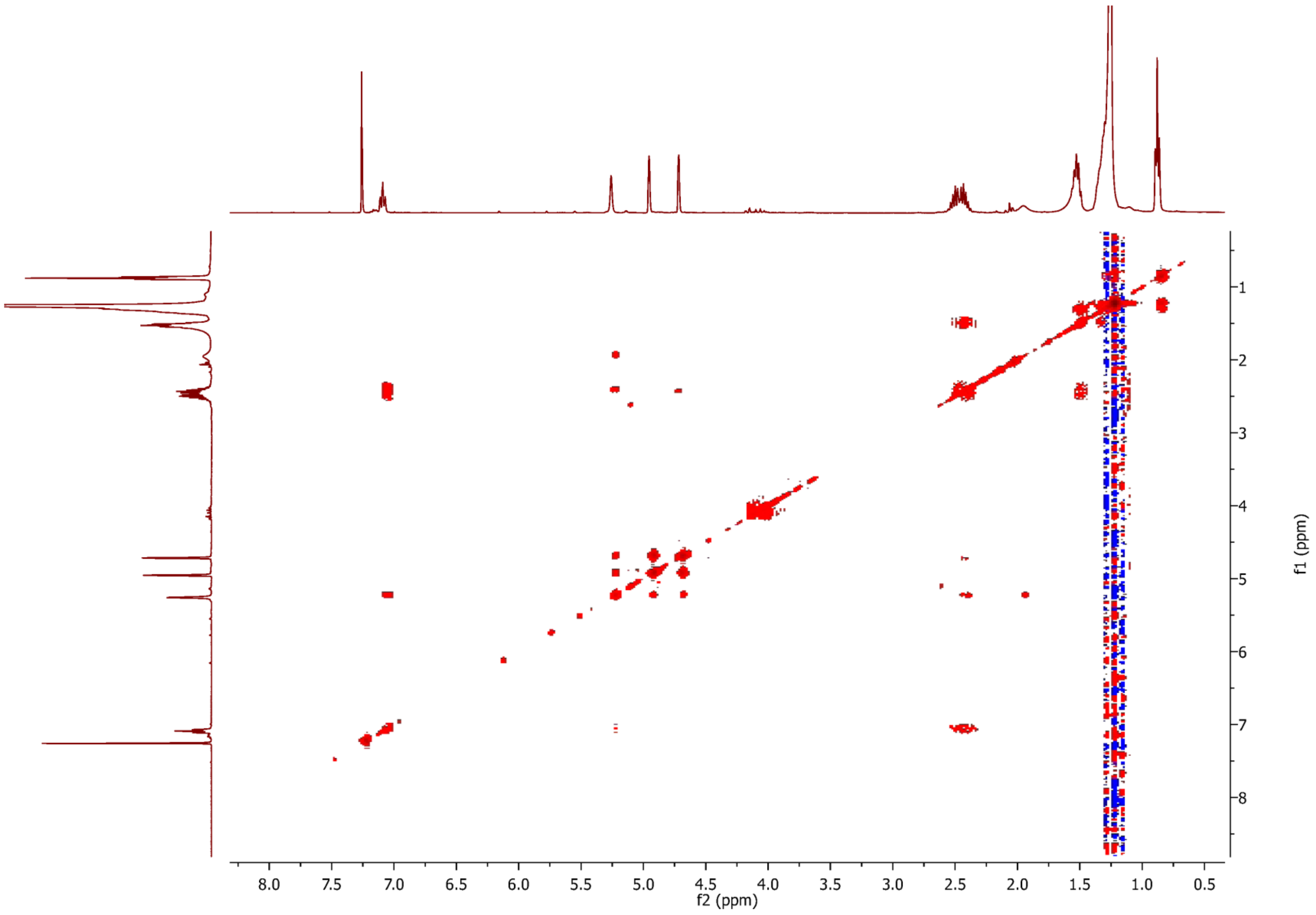

Figure S6: gHSQCAD NMR spectrum of isozuihoenalide 1.

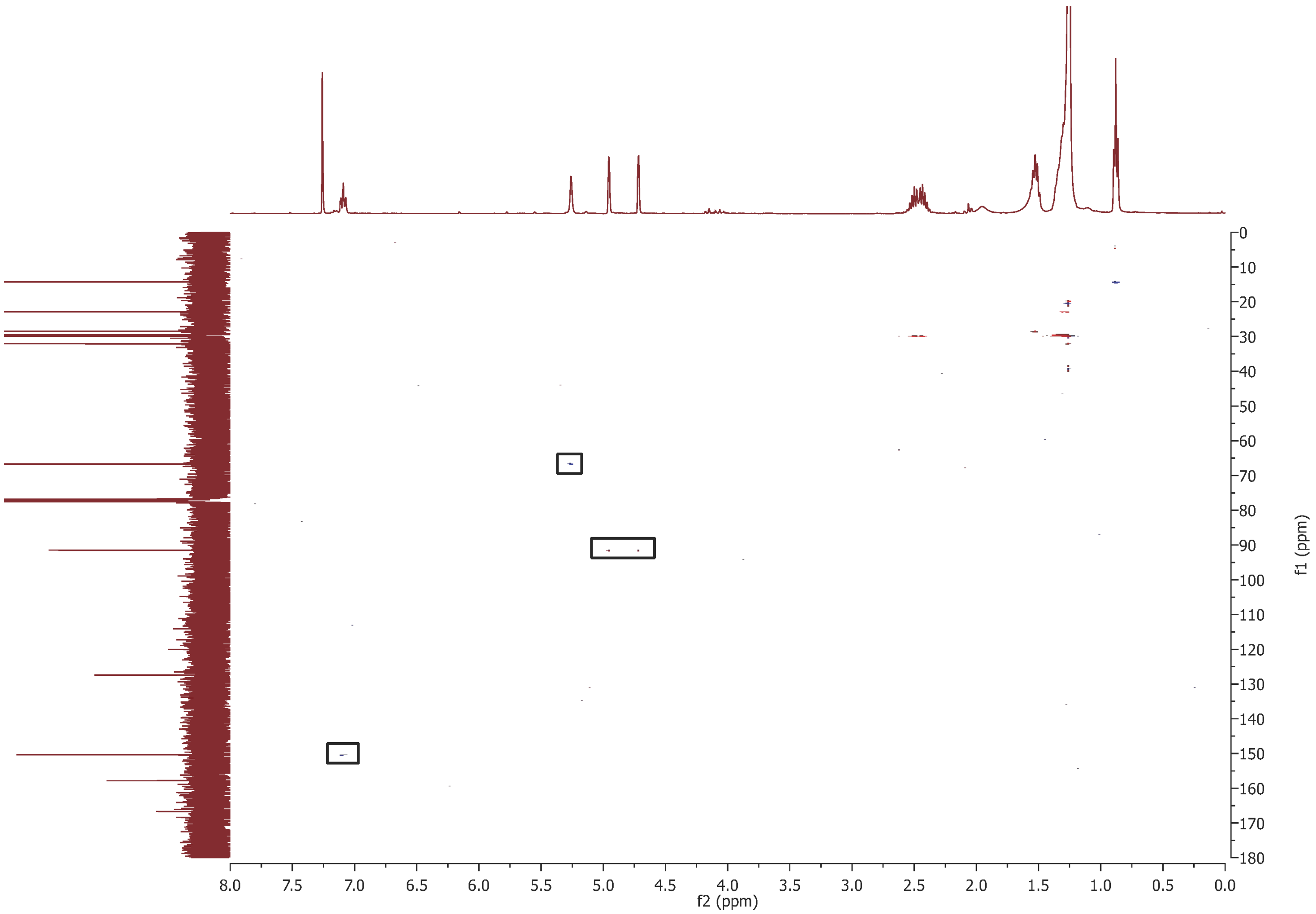

Figure S7: gHMBC NMR spectrum of isozuihoenalide 1.

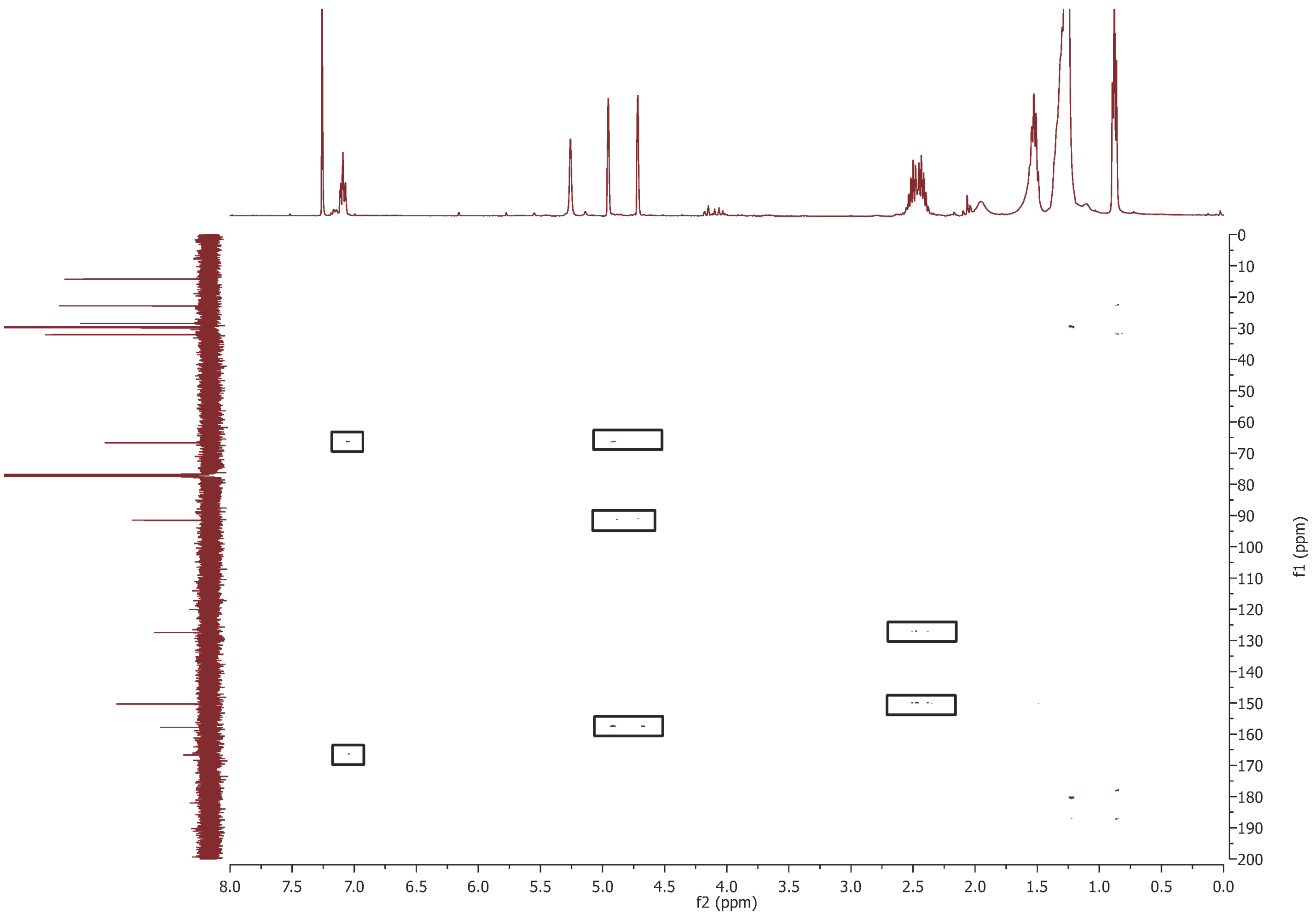

Figure S8: Experimental and predicted ECD spectra of isozuihoenalide 1**.**

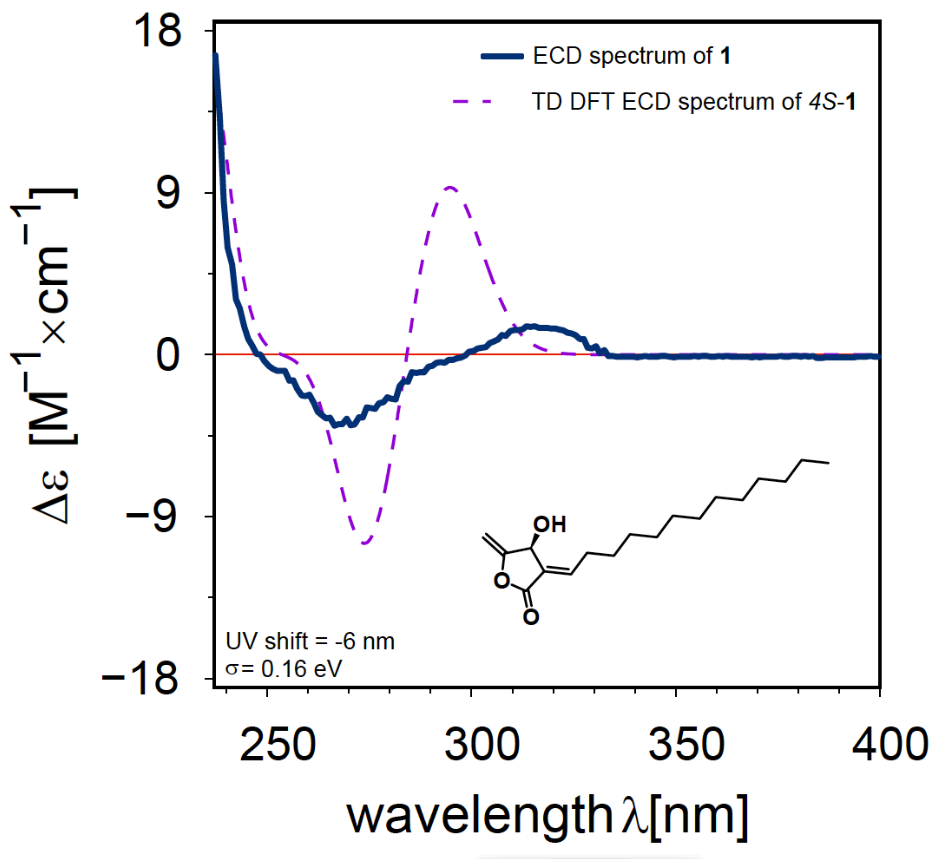

Figure S9: MS1 and MS2 spectra of γ-lactone 2 from sapwood extract (RT= 15.64 min, Supplementary Fig S1-d).

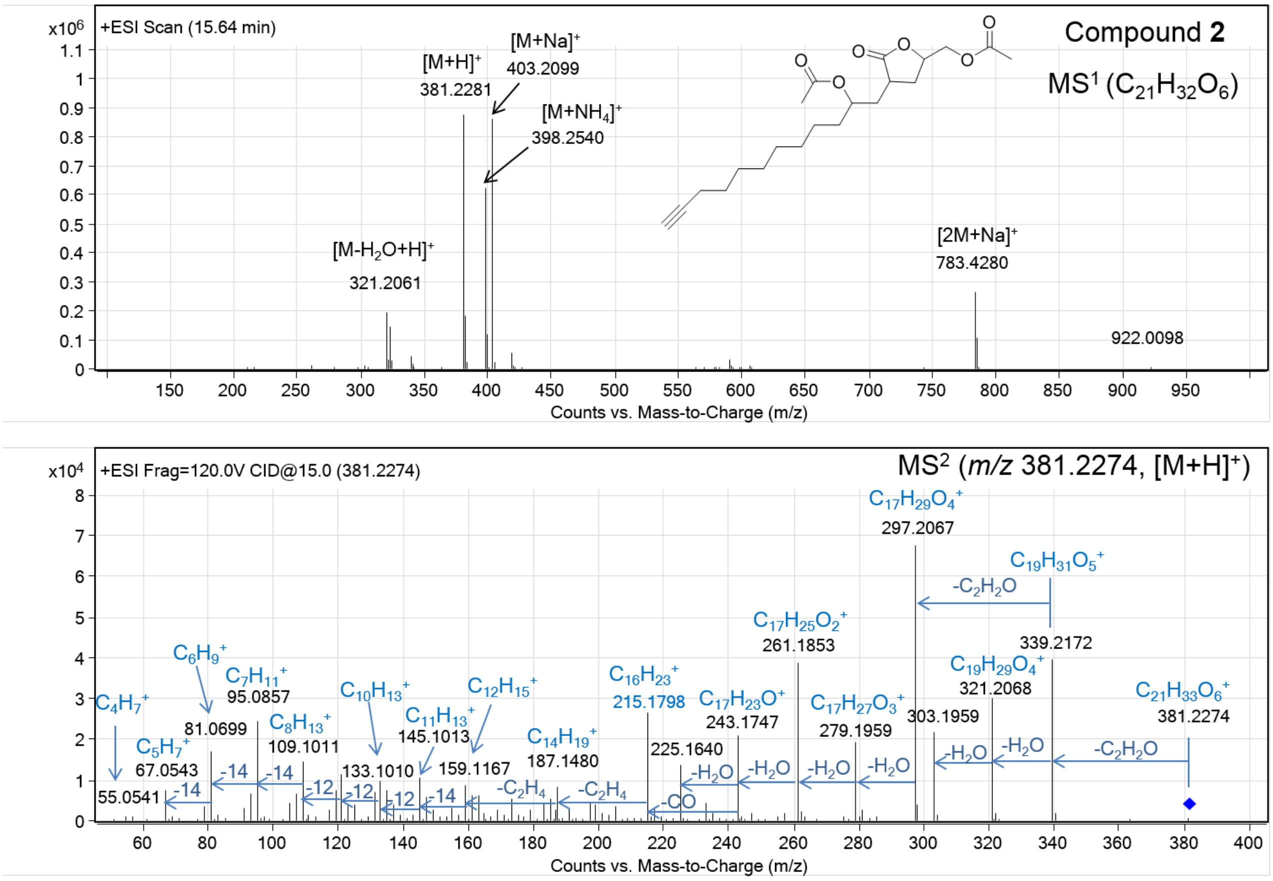

Figure S10: MS1 and MS2 spectra of γ-lactone 3 from sapwood extract (RT= 17.74 min, Supplementary Fig S1-d).

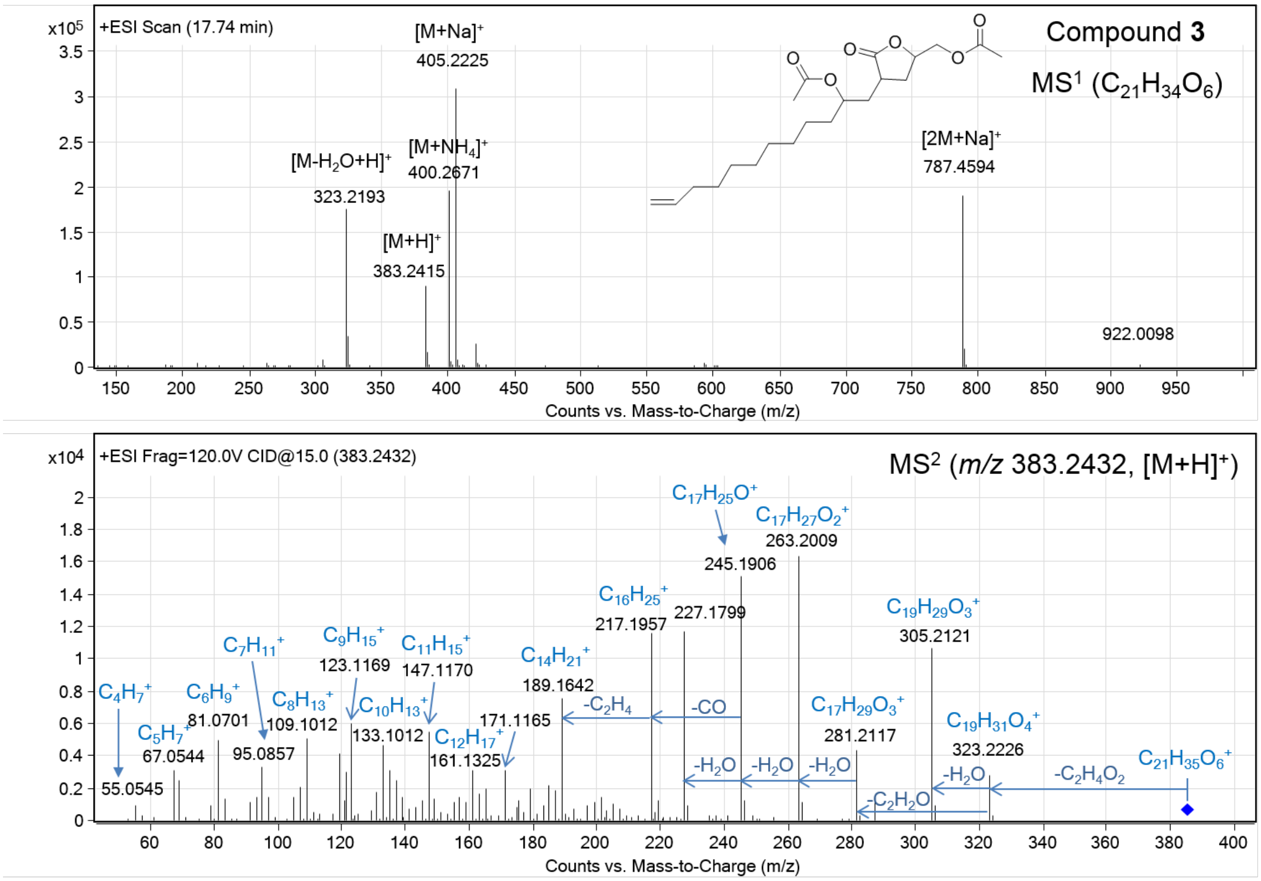

Table S2: NMR data of γ-lactones 2 and 3.

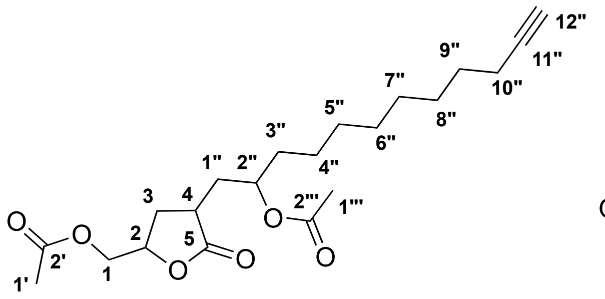

(2)

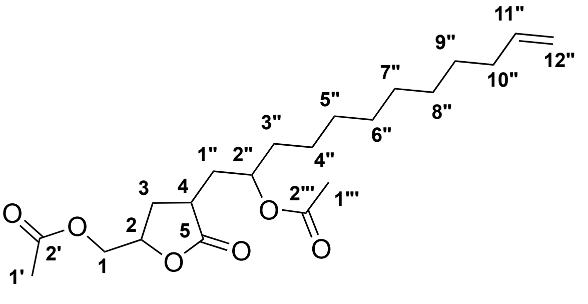

(3)

$[\alpha]^{25}$= +35.0 (c= 0.50, EtOAc)

$[\alpha]^{25}$= +34.8 (c= 0.40, EtOAc)

N°	^{13}C (δ)		^{1}H (δ)	
	γ-lactone **2**	γ-lactone **3**	γ-lactone **2**	γ-lactone **3**
1	65.17 (CH2)	65.21 (CH2)	4.24 (1H, dd, *J* = 11.9, 1.4 Hz, H-1a)<br>4.02 (1H, dd, *J* = 11.8, 1.4 Hz, H-1b)	4.26 (1H, dd, *J* = 11.9, 3.6 Hz, H-1a)<br>4.04 (1H, dd, *J* = 11.9, 6.4 Hz, H-1b)
2	69.28 (CH)	69.27 (CH)	5.13 (1H, br s)	
3	32.14 (CH2)	32.16 (CH2)	2.33 (1H, t, *J* = 11.7 Hz, H-3a)<br>1.59-1.38 (1H, m, H-3b)	2.34 (1H, ddd, *J* = 14.7, 10.5, 3.3 Hz, H-3a)<br>1.65-1.42 (5H, m, H-3b)
4	37.92 (CH)	37.95 (CH)	2.60 (1H, m)	
5	178.10 (C)	178.18 (C)		
1’	21.15 (CH3)		2.08 (3H, s)	
2’	170.71 (C)	170.77(C)		
1”	35.75 (CH2)	35.78 (CH2)	2.53 (1H, m, H-1”a)<br>1.49 (1H, m, H-1”b)	2.53 (1H, m, H-1”a)<br>1.49 (1H, m, H-1”b)
2”	79.21 (CH)	79.28 (CH)	4.33 (1H, br s)	4.35 (1H, m)
3”	35.51 (CH2)	35.55	1.72 (1H, m, H-3”a)<br>1.57 (1H, m, H-3”b)	1.72 (1H, m, H-3”a)<br>1.57 (1H, m, H-3”b)
4”	25.35 (CH2)		1.45-1.25 (10H, br s, H-4”–8”)	1.42-1.25 (10H, br s, H-4”–8’)
5”	29.5-28.5 (CH2)		1.45-1.25 (10H, br s, H-4”–8”)	1.42-1.25 (10H, br s, H-4”–8’)
6”-7”	29.5-28.5 (CH2)		1.45-1.25 (10H, br s, H-4”–8”)	1.42-1.25 (10H, br s, H-4”–8’)
8”	29.5-28.5 (CH2)		1.45-1.25 (10H, br s, H-4”–8”)	1.42-1.25 (10H, br s, H-4”–8’)
9”	29.5-28.5 (CH2)		1.59-1.38 (2H, m)	1.65-1.42 (2H, m)
10”	18.4 (CH2)	33.93 (CH2)	2.17 (2H, t, *J* = 7.06 Hz)	2.05 (2H, m)
11”	84.81 (C)	139.32 (CH)		5.82 (1H, m)
12”	68.23 (CH)	114.30 (CH2)	1.93 (1H, s)	5.00 (1H, d, *J* = 17.7, H_E-12”)

				4.92 (1H, d, *J* = 10.1, H_Z-12”)
1”’	20.8 (CH3)		2.06 (3H, s)	
2”’	170.76 (C)	170.82(C)		

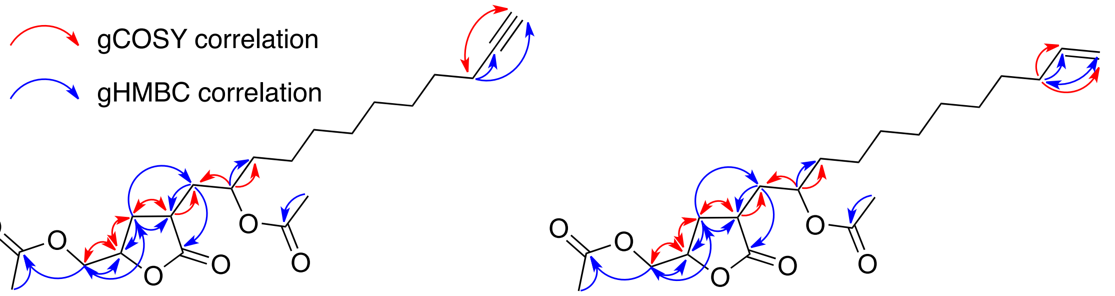

N°	gCOSY (^{1}H-^{1}H)		gHMBC (^{1}H-^{13}C)	
	γ-lactone **2**	γ-lactone **3**	γ-lactone **2**	γ-lactone **3**
1	H2		C2 - C3 - C2’	
2	H1 - H3			
3	H2 - H4 - H1”		C1 - C2 - C4 - C1”	
4	H3 - H1”			
1’			C2’	
1”			C4 - C5	
2”	H1” - H3”		C3”	
10”	H12”	H11” - H12”	C11” - C12”	C11” - C12”
12”	H10”			C10”
1”’				C2”’

Figure S11: 1H NMR spectrum of γ-lactone 2.

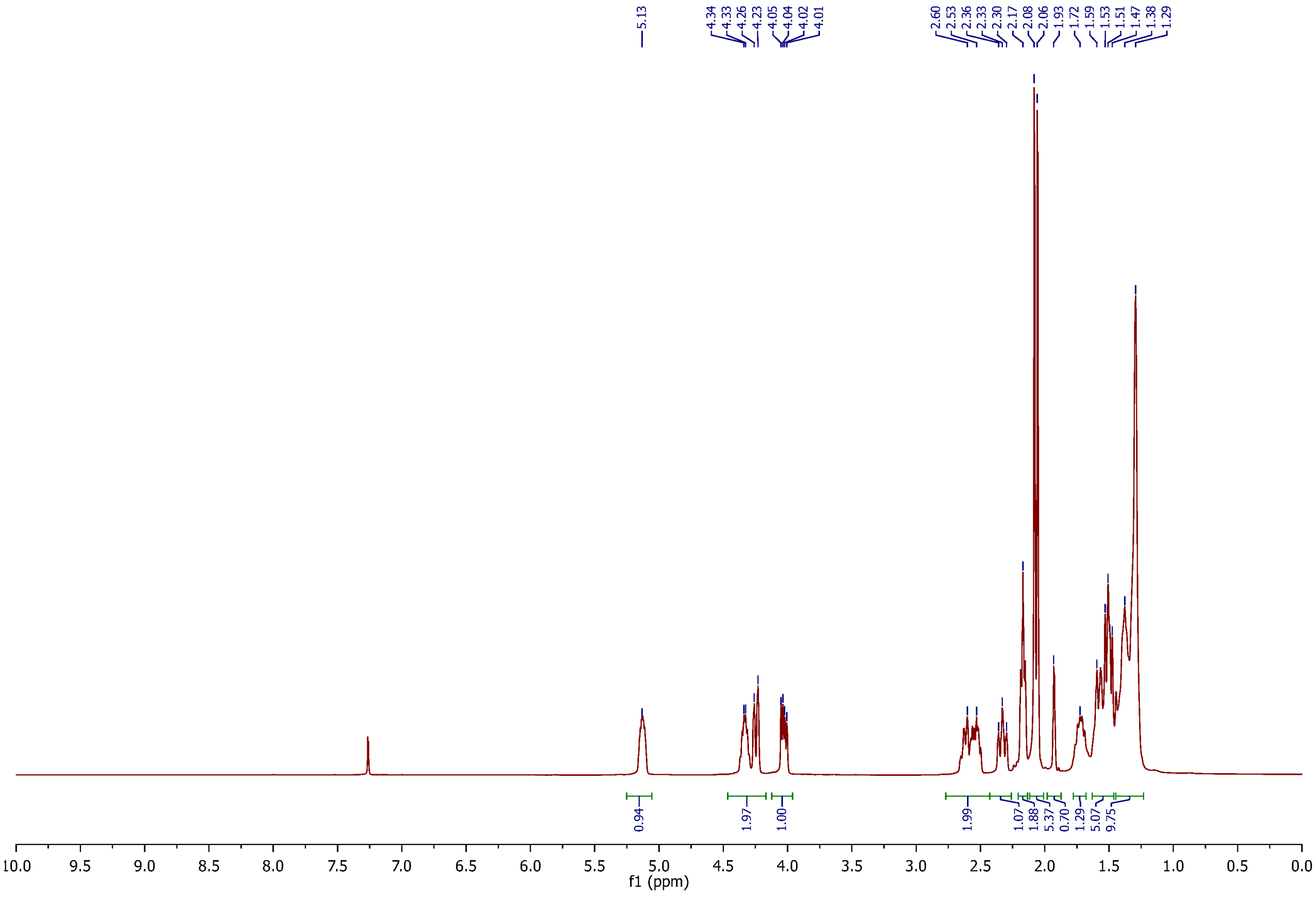

Figure S12: 13C NMR spectrum of γ-lactone 2.

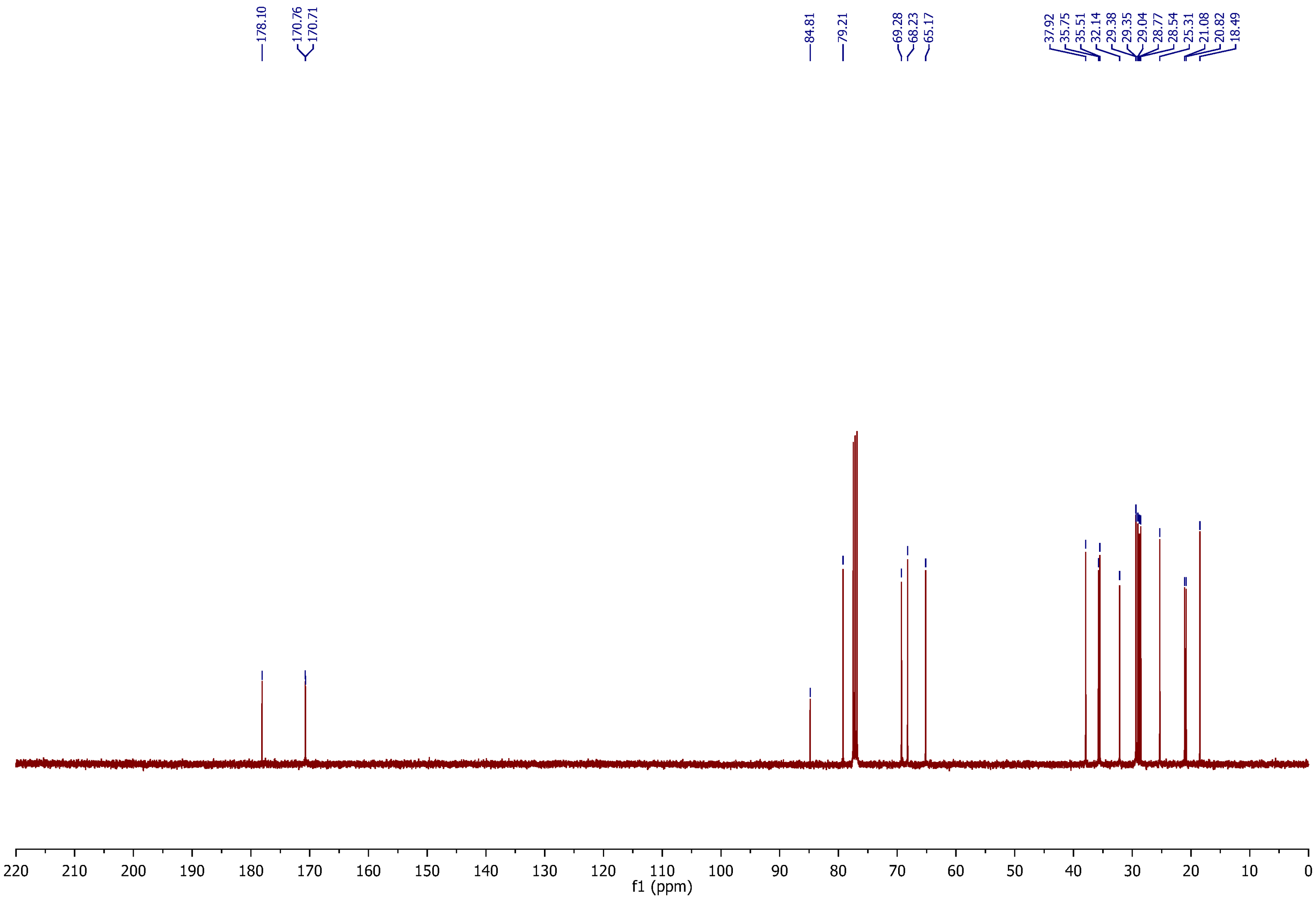

Figure S13: gCOSY NMR spectrum of γ-lactone 2.

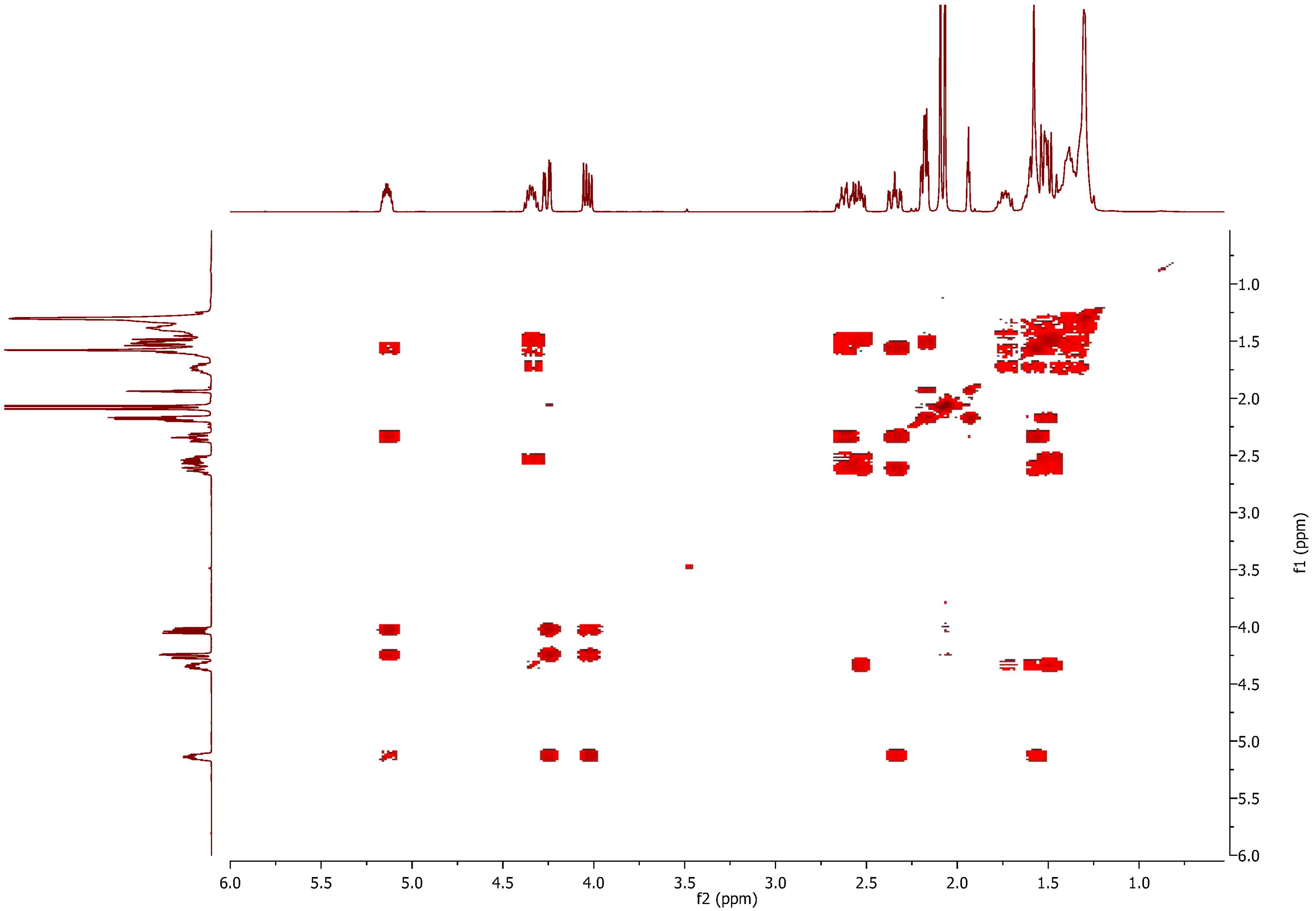

Figure S14: gHSQCAD NMR spectrum of γ-lactone 2.

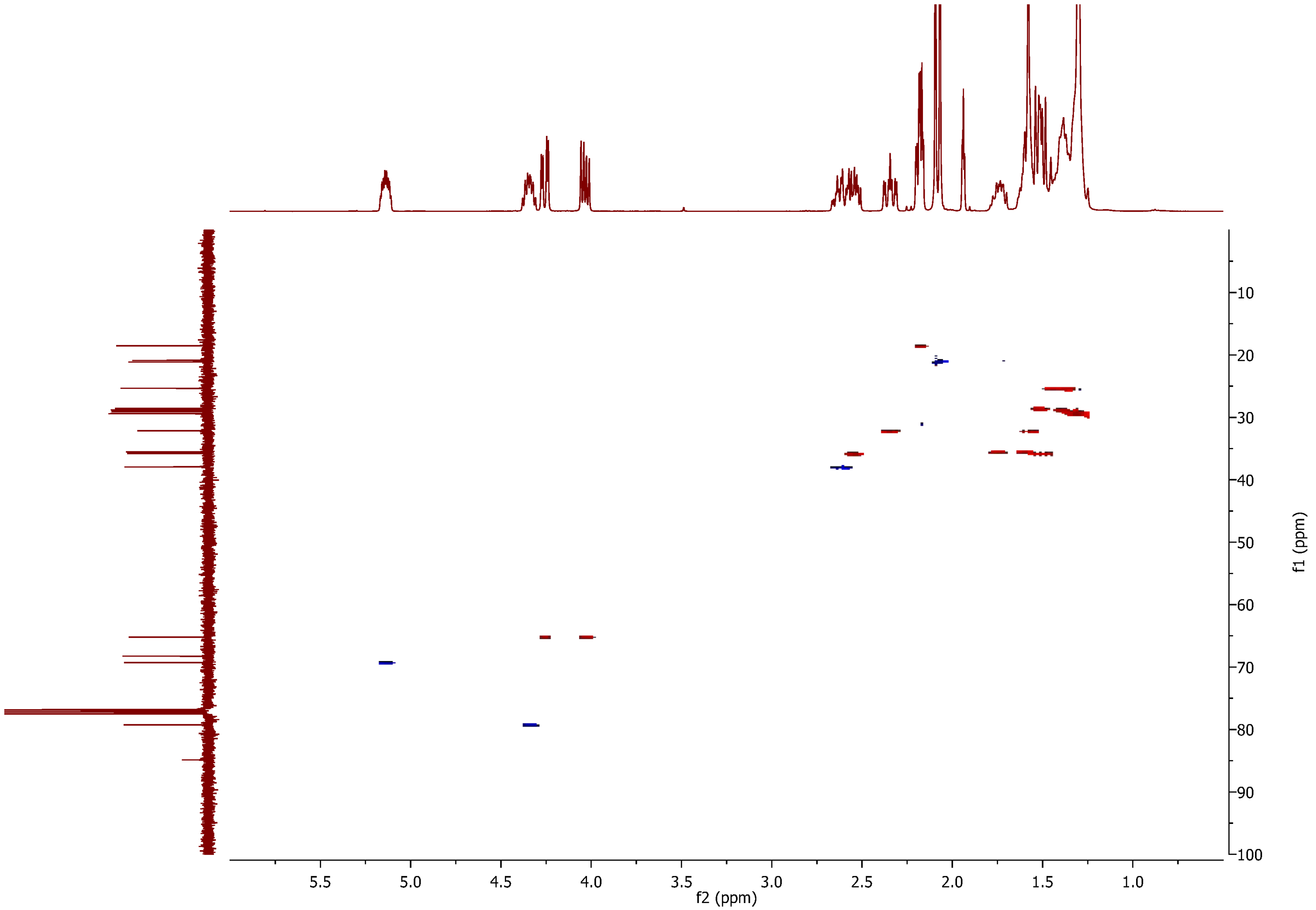

Figure S15: gHMBC NMR spectrum of γ-lactone 2.

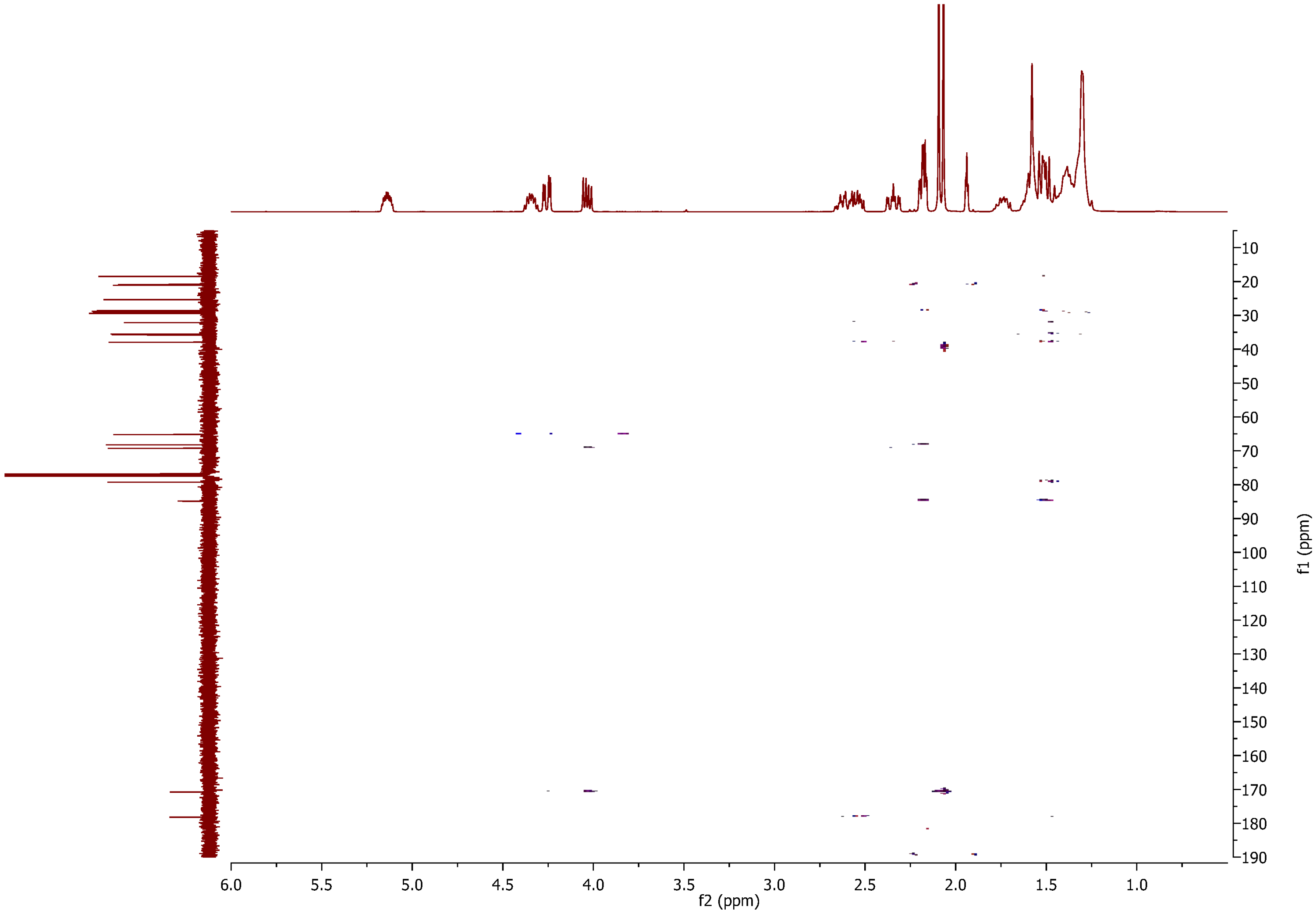

Figure S16: 1H NMR spectrum of γ-lactone 3.

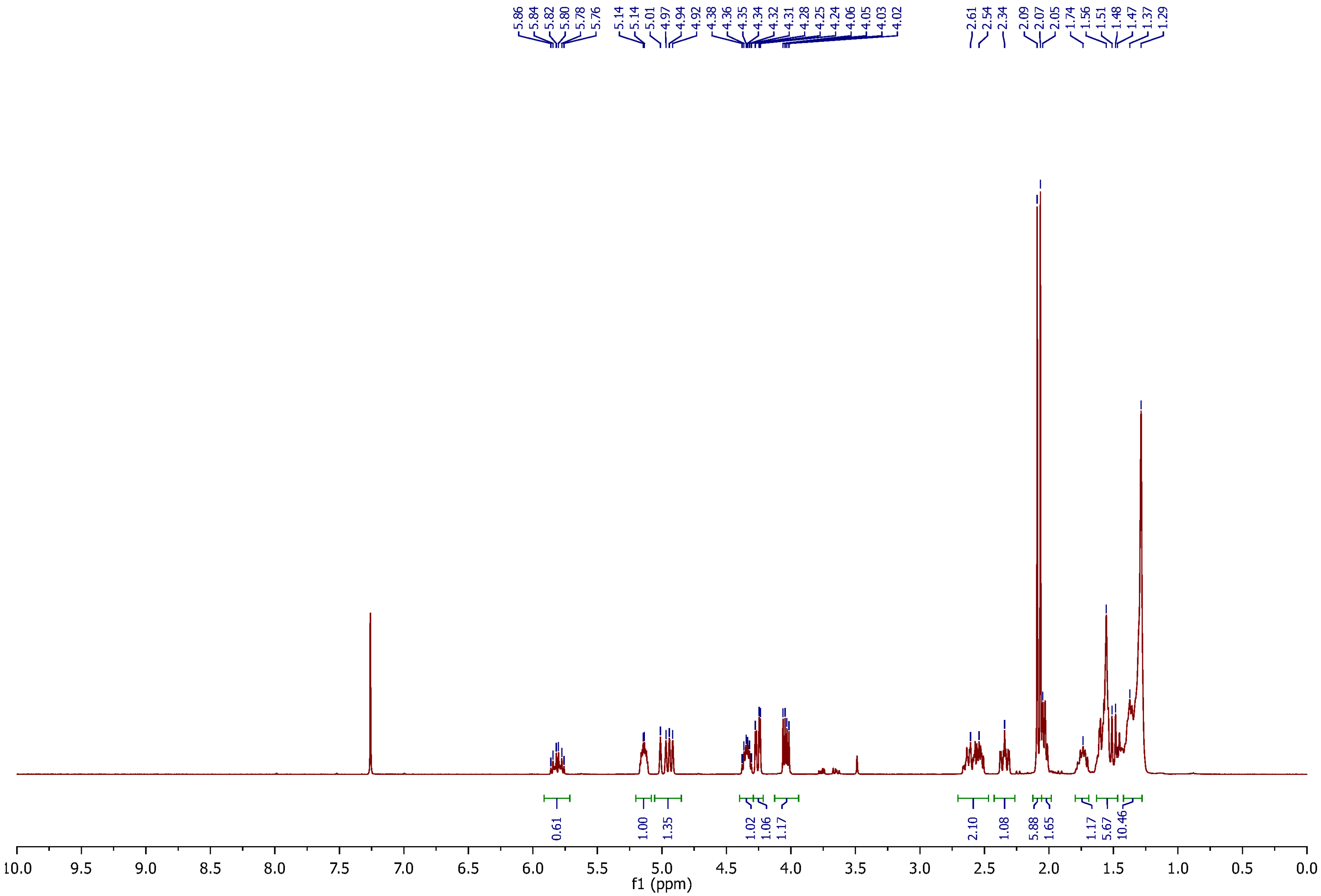

Figure S17: 13C NMR spectrum of γ-lactone 3.

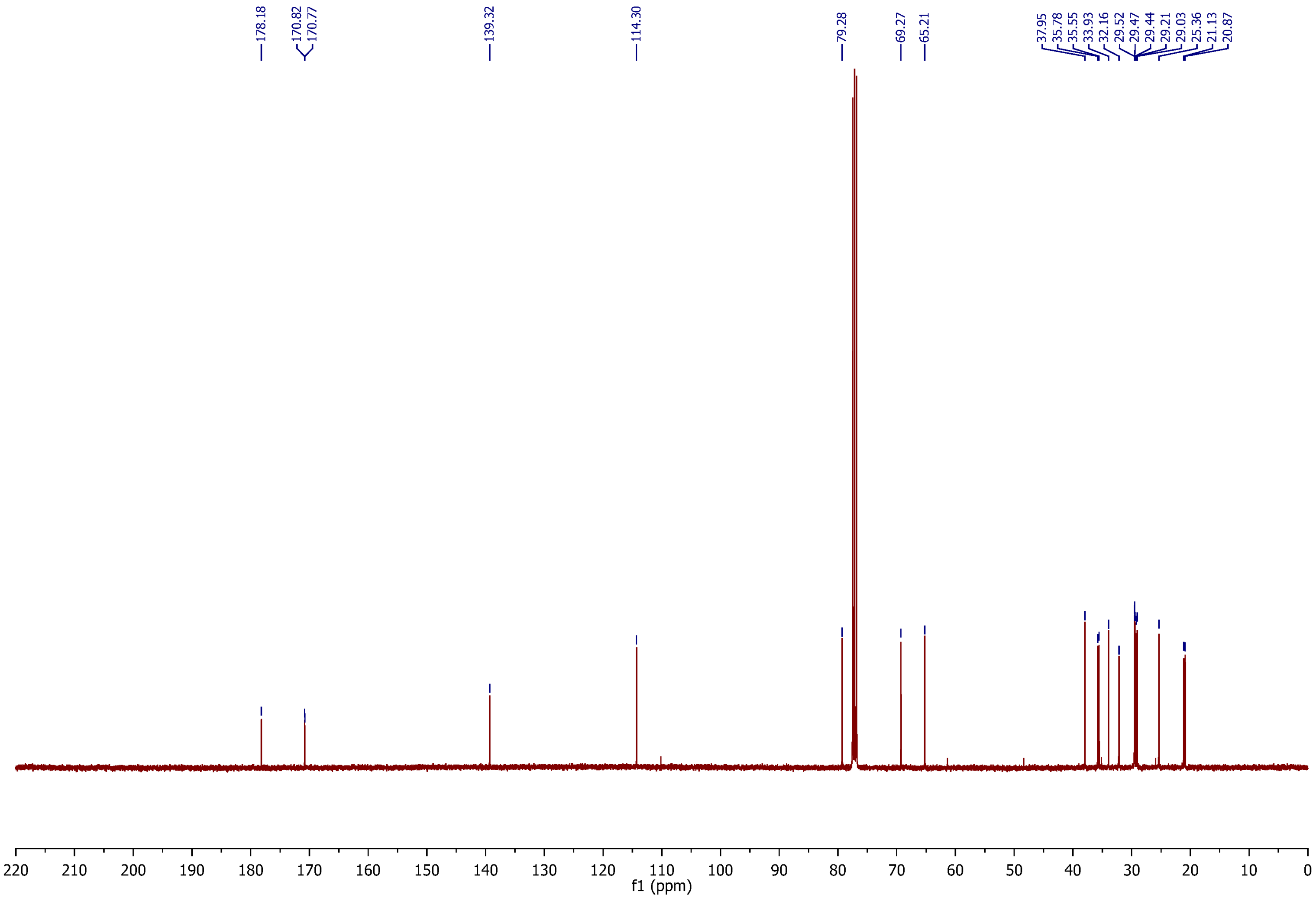

Figure S18: gCOSY NMR spectrum of γ-lactone 3.

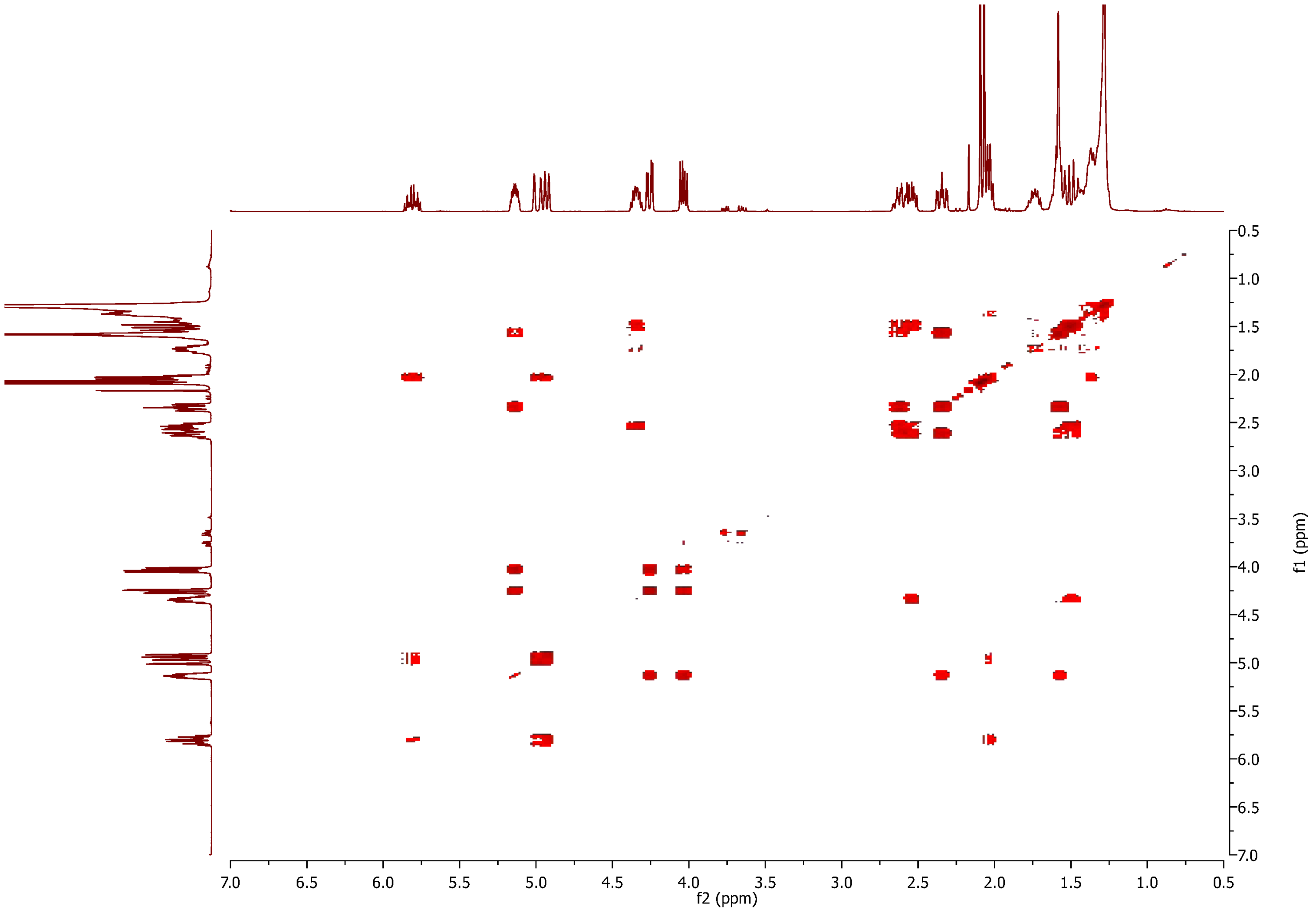

Figure S19: gHSQCAD NMR spectrum of γ-lactone 3.

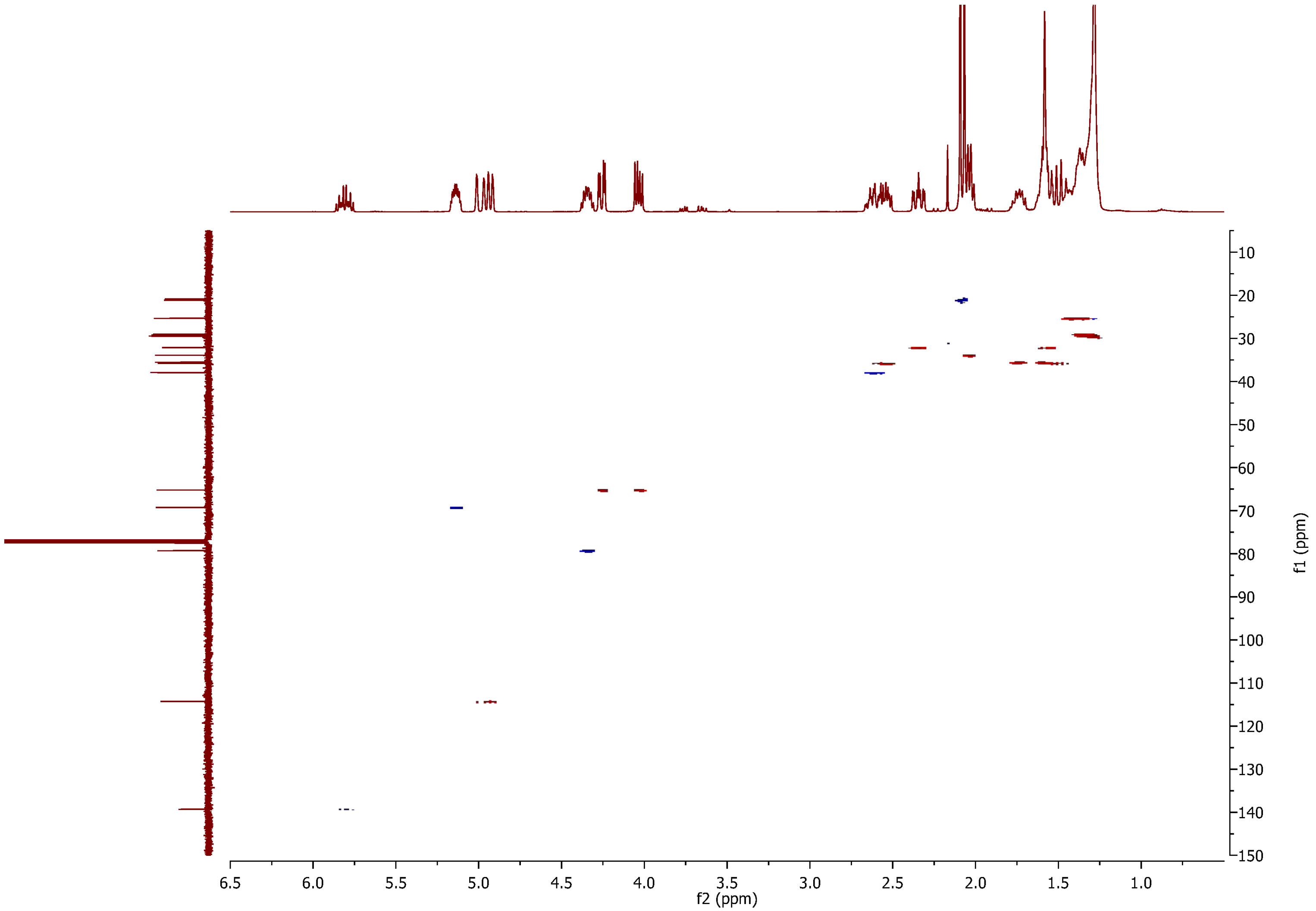

Figure S20: gHMBC NMR spectrum of γ-lactone 3.

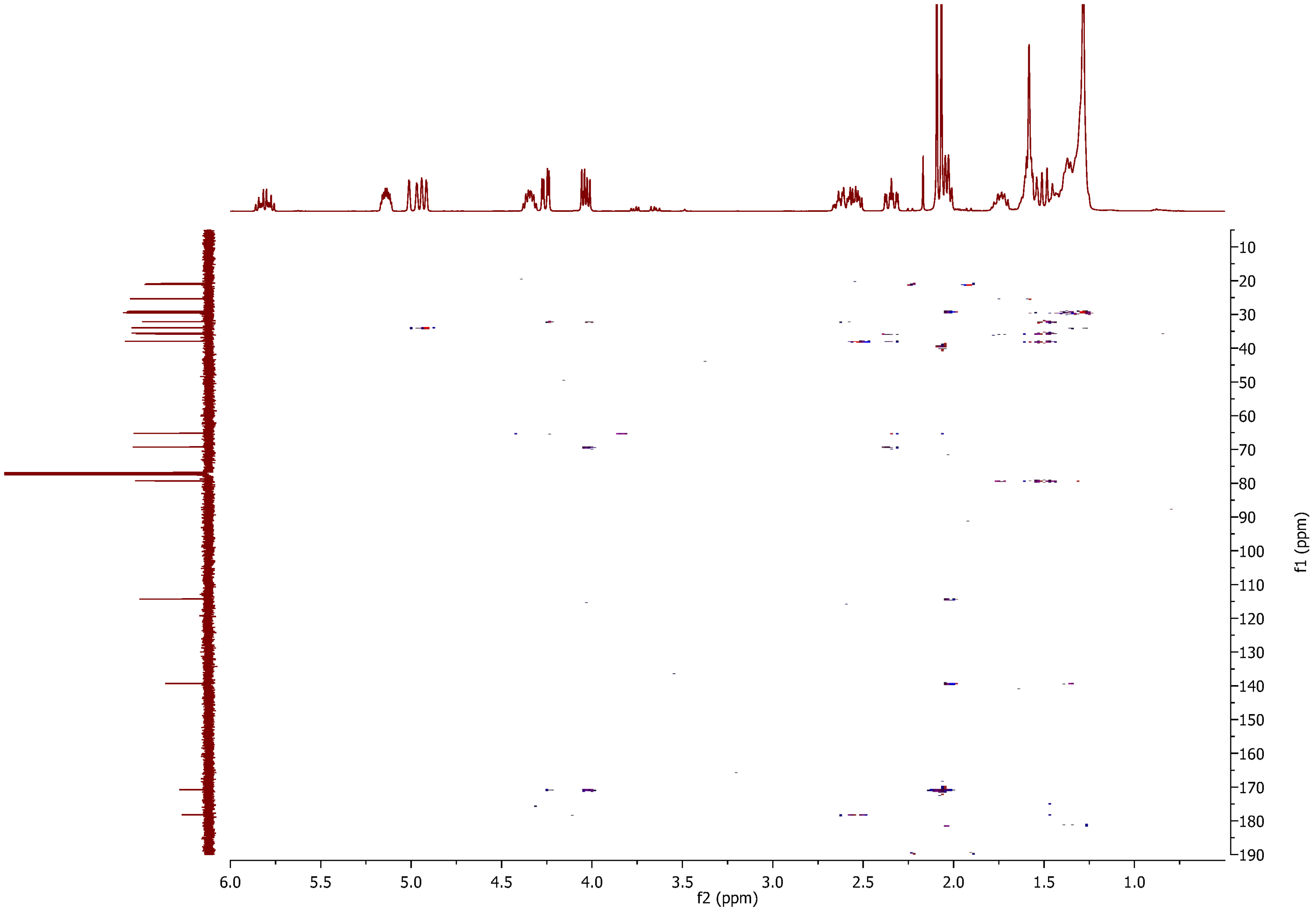

Figure S21: Experimental and predicted ECD spectra of γ-lactone 2**.**

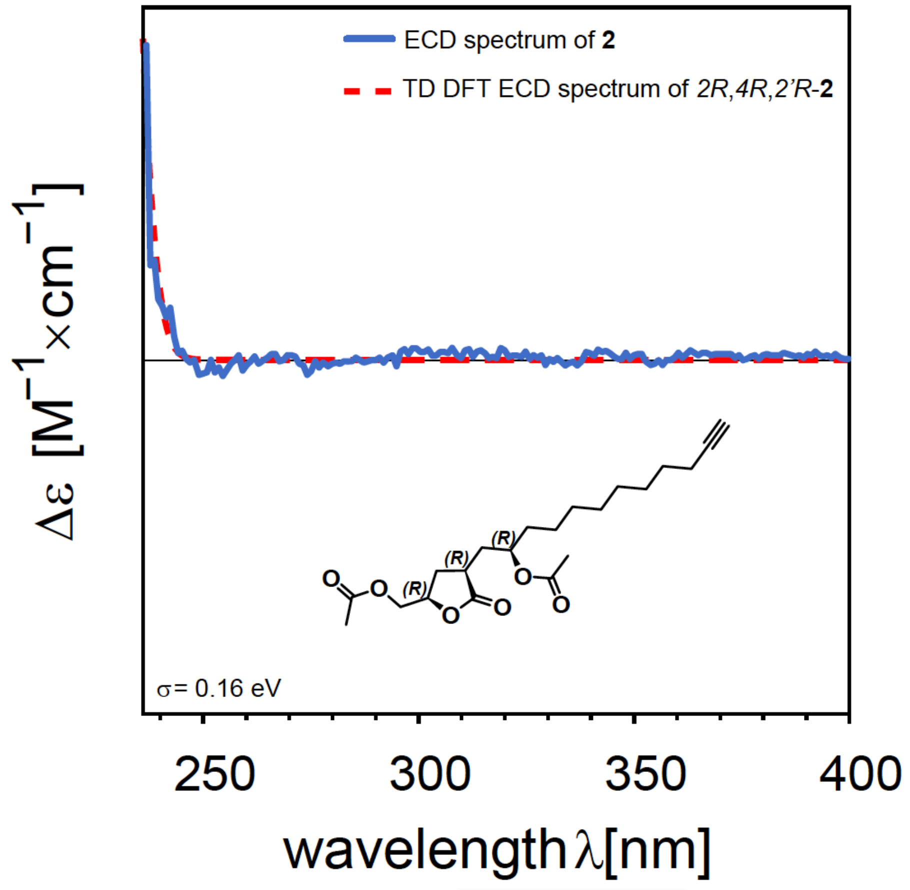

Table S3: Extraction yields of γ-lactones from roots, sapwood, bark, and heartwood extracts by 1H NMR quantification.

**Material**		**Extraction yield (%)[a]**			
	**Roots**	**Sapwood**	**Heartwood**	**Bark**	**Leaf**
crude extract	8.7	8.1	9.2	1.7	3.7
isozuihoenalide **1**	2.1	1.9	nd.	un.	un.
γ-lactones **2-3**	6.0	5.3	nd.	0.2	un.
γ-lactones **4-5**	nd.	nd.	8.1	un.	un.

[a]Measurements were performed on *S. rubra* (individual Sr1) in $CDCl_3$ using 1,2,4,5-tetrachloronitrobenzene as internal standard. nd.: not detected. un.: unknown (cannot be determined due to the overlap with interference peaks).

Figure S22: 1H NMR spectra of bark extracts. Red dots show proton ^{1}H NMR signals used for the quantification of γ-lactones **2**-**3**.

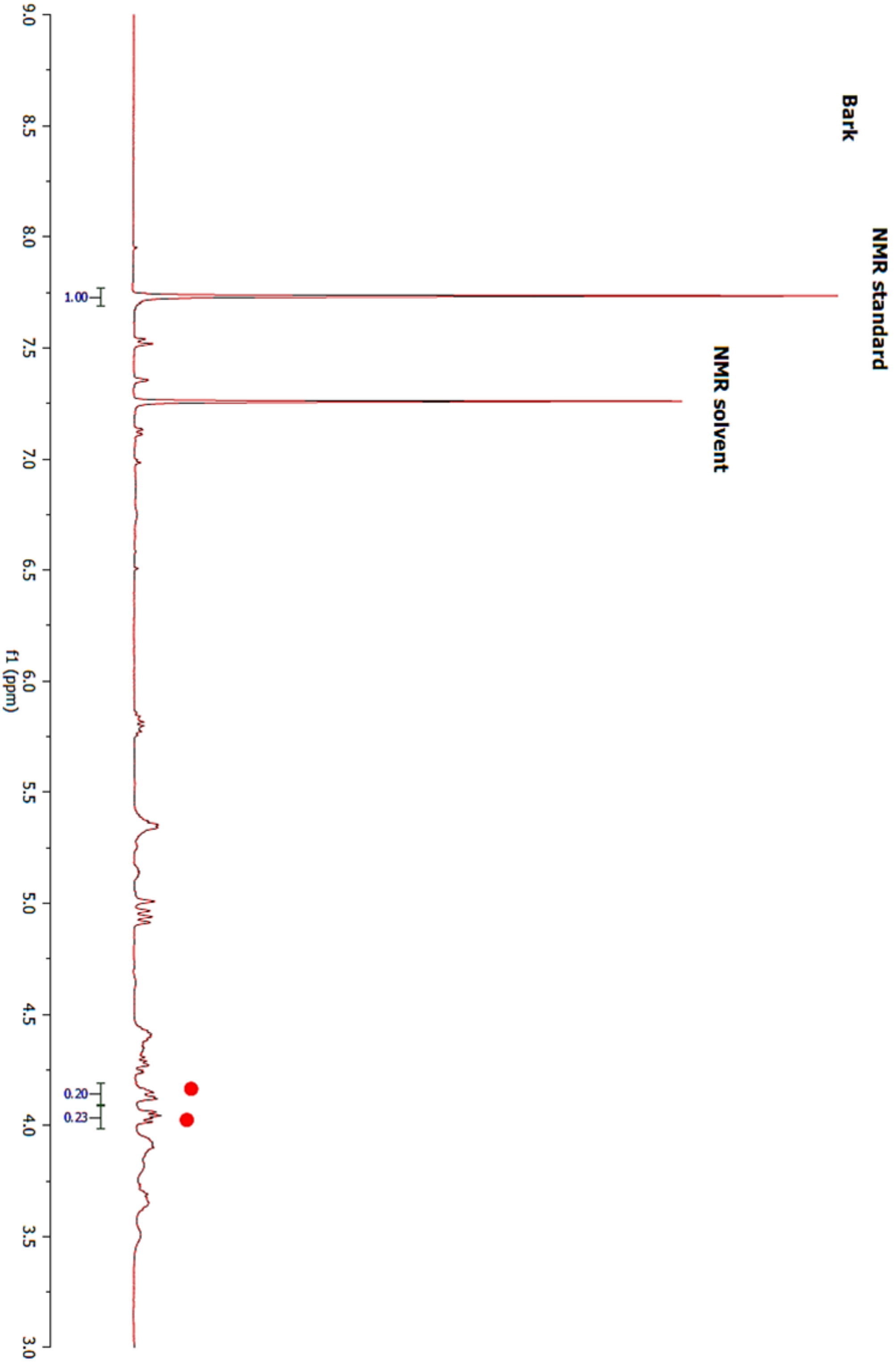

Figure S23: 1H NMR spectra of sapwood extracts. Black and red dots show proton ^{1}H NMR signals used for the quantification of γ-lactones **1** and **2**-**3**.

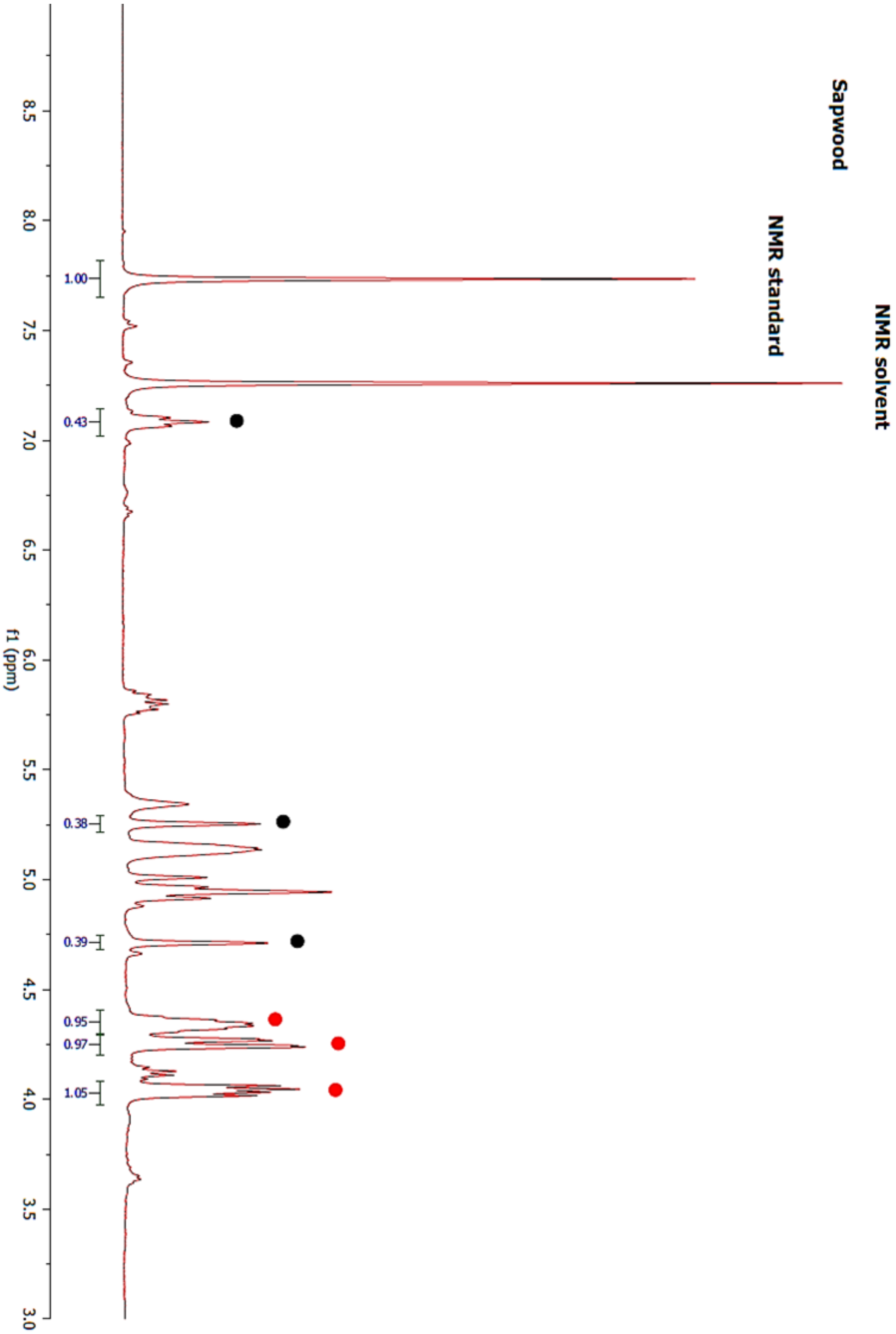

Figure S24: 1H NMR spectra of heartwood extracts. Black and blue dots show proton ^{1}H NMR signals used for the quantification of γ-lactones **1** and **4-5**.

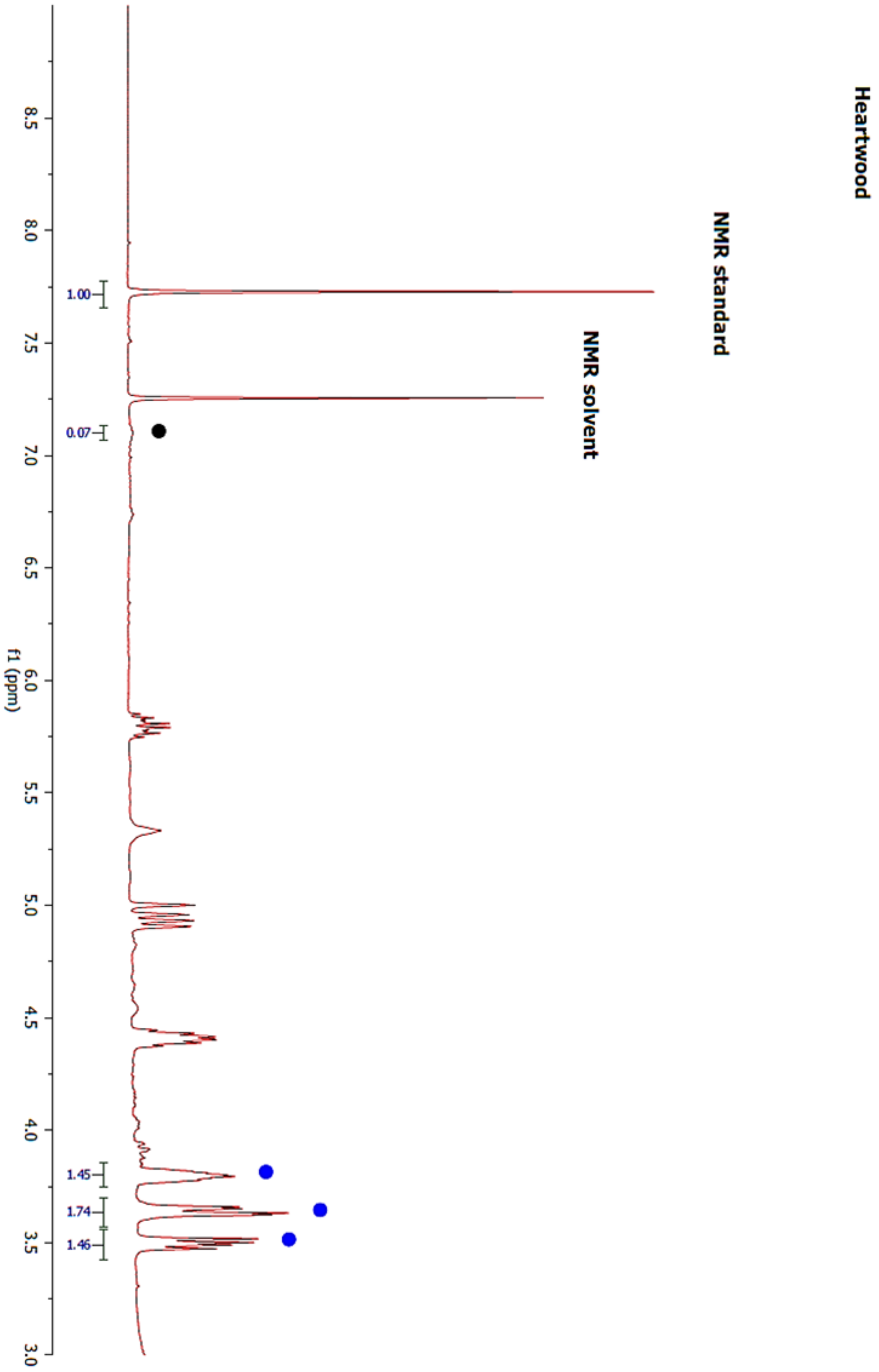

Figure S25: 1H NMR spectra of root extracts. Black and red dots show proton 1H NMR signals used for the quantification of γ-lactones **1** and **2**-**3**.

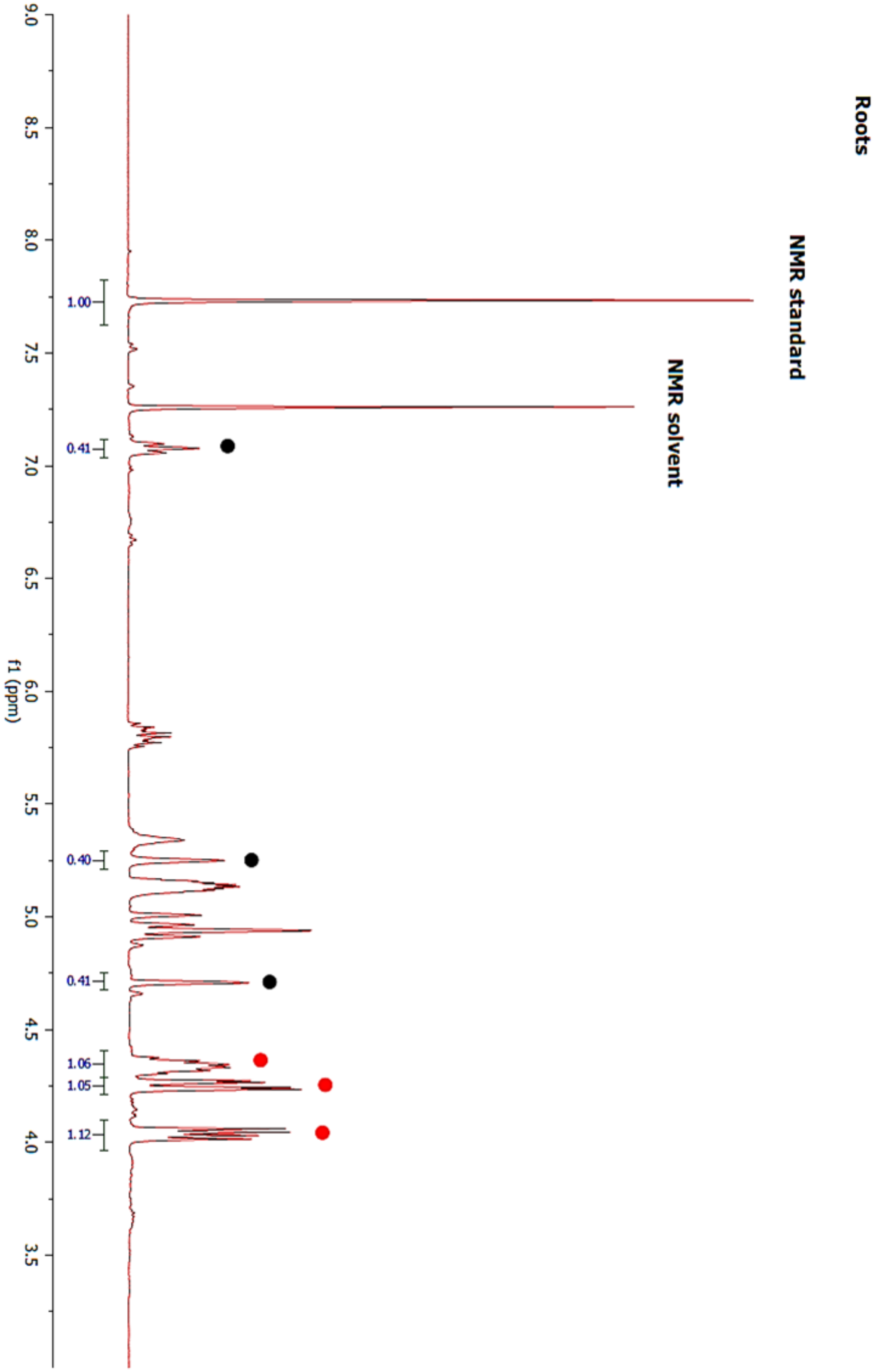

Figure S26: 1H NMR spectra of leaf extracts. Black dot shows proton ^{1}H NMR signals used for the quantification of γ-lactone **1**.

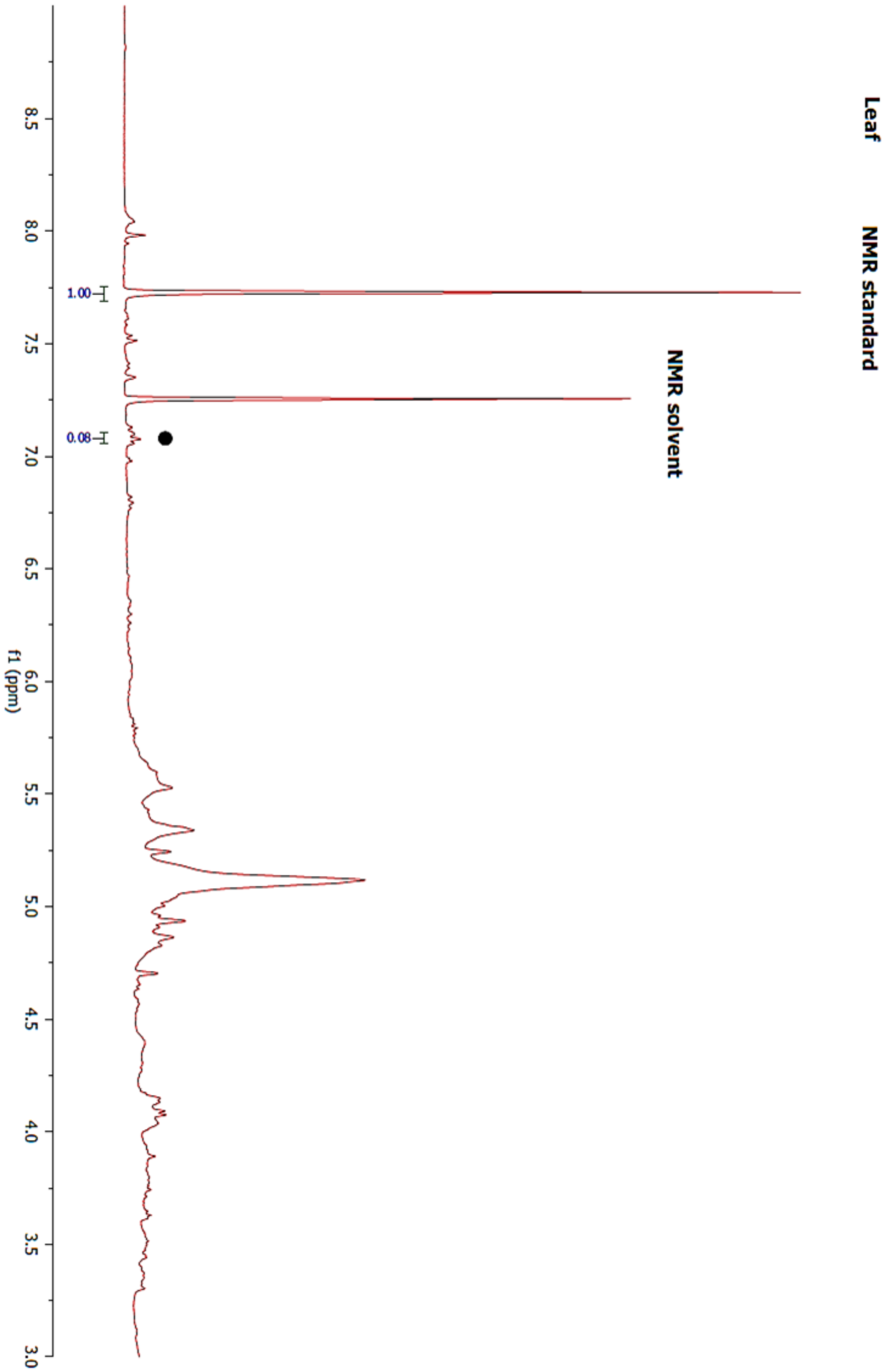

Figure S27: MS1 and MS2 spectra of protonated ion at m/z 279.1961 from sapwood extract (RT= 16.12 min, Supplementary Fig. S1-d). Assigned as γ-lactone **6**.

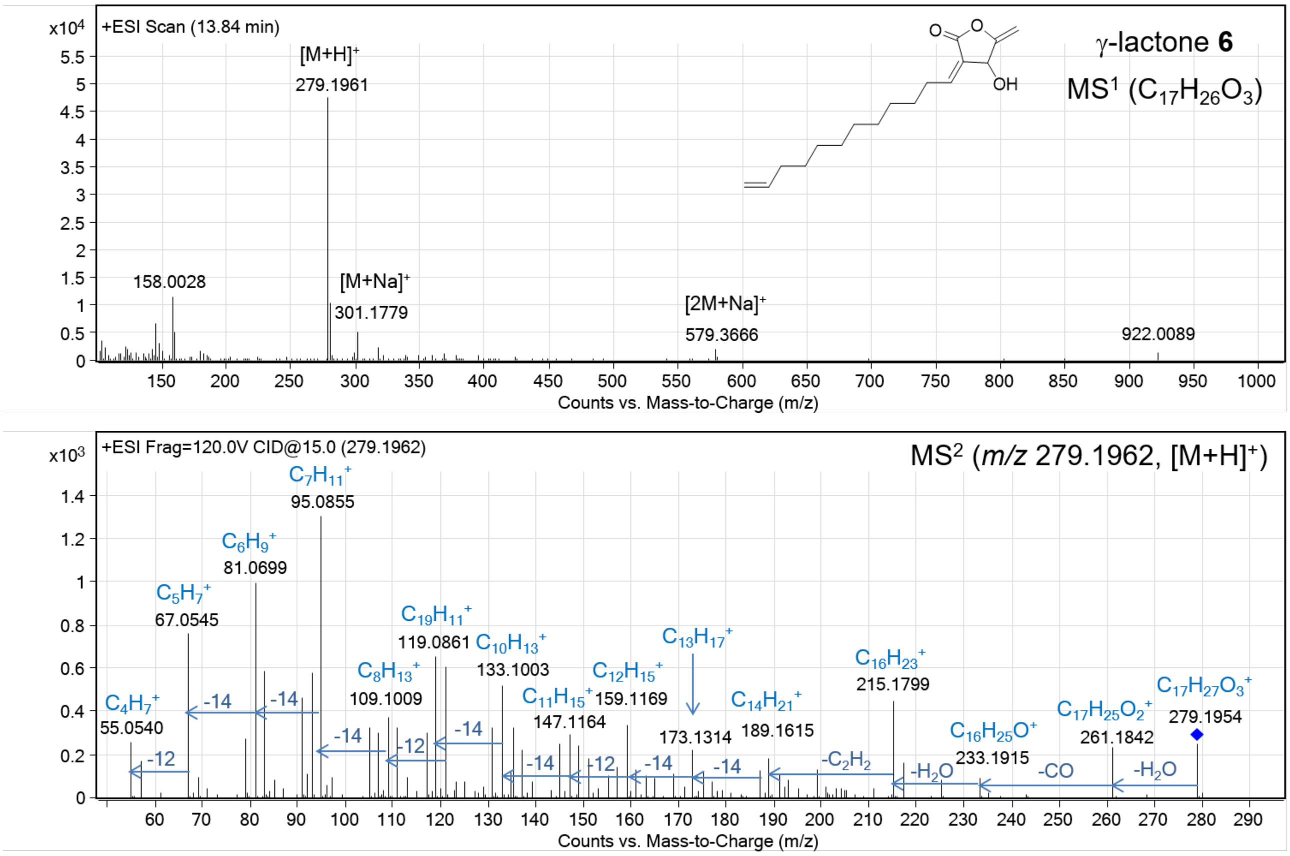

Figure S28: MS1 and MS2 spectra of protonated ion at m/z 321.2064 from sapwood extract (RT= 13.84 min, Supplementary Fig. S1-d). Assigned as γ-lactone **8**.

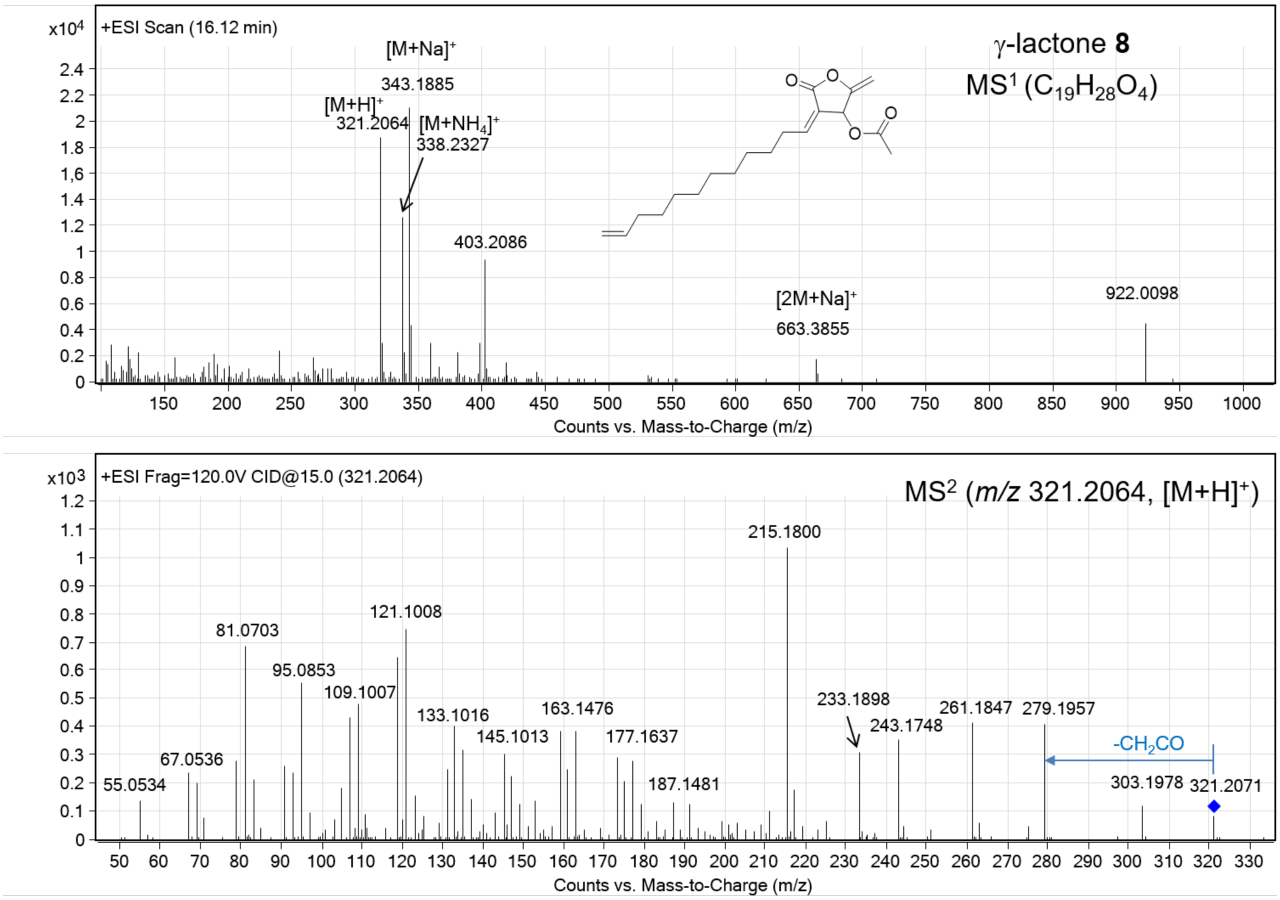

Figure S29: TOF-SIMS tandem MS imaging and product ion peak attributions. (a) Structure of γ-lactones **6-9**. (b) Optical image of transition wood surface. (c) Total MS^1 ion (TIC) image (gray scale). (d) MS^1 image of Ca^+ (*m/z* 40). (e) MS^1 image of lignin ions. (f) MS^2 TIC image of *m/z* 279 precursor ions. The product ion attributions, MS/MS spectrum of the precursor ions at *m/z* 279 shown in panel (l), support the structure of γ-lactone **6**. (g) MS^2 TIC image of *m/z* 281 precursor ions. The product ion attributions, MS/MS spectrum of the precursor ions at *m/z* 281 shown in panel (m), support the structure of γ-lactone **7**. (h) MS^1 image of *m/z* 297 ions (γ-lactone **4**). (i) MS^1 image of *m/z* 299 ions (γ-lactone **5**). (j) MS^2 TIC image of *m/z* 321 precursor ions. The product ion attributions, MS/MS spectrum of the precursor ions at *m/z* 321 shown in panel (n), support the structure of γ-lactone **8**. (k) MS^2 TIC image of *m/z* 323 precursor ions. The product ion attributions, MS/MS spectrum of precursor ions at *m/z* 323 shown in panel (n), support the structure of γ-lactone **9**. Each ion image has a field-of-view of 150 µm x 150 µm with a pixel dimension of 586 nm. Lignin fragments used were: $C_8H_9O_2^+$ (*m/z* 137.06), $C_9H_{11}O_2^+$ (*m/z* 151.07), $C_8H_8O_3^+$ (*m/z* 152.05), $C_8H_9O_3^+$ (*m/z* 153.06), $C_9H_9O_3^+$ (*m/z* 165.06), $C_9H_{11}O_3^+$ (*m/z* 167.07) and $C_{10}H_{13}O_3^+$ (*m/z* 181.08), $C_{11}H_9O_3^+$ (*m/z* 189.06).

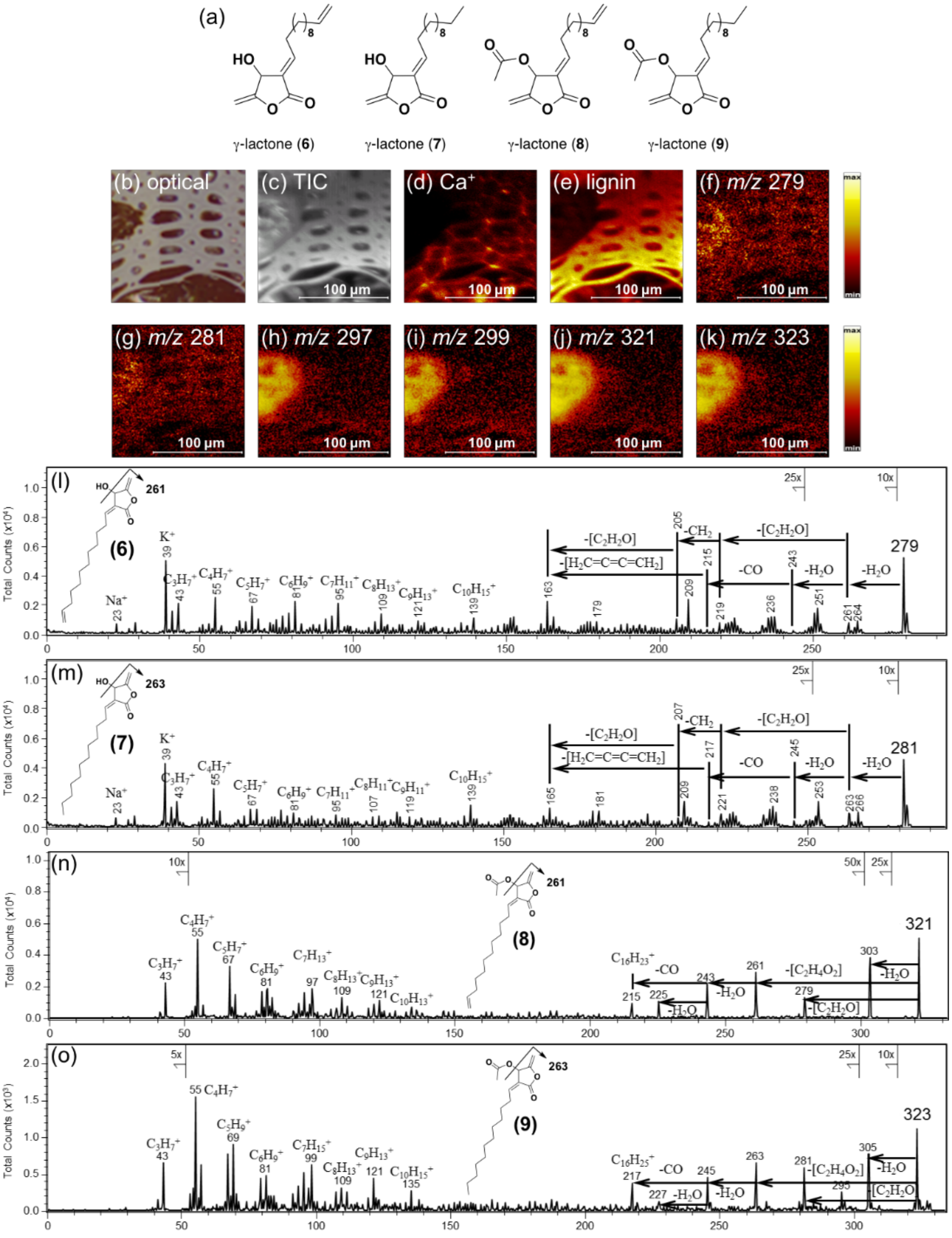
(a)
HO
O
8
γ-lactone (6)
γ-lactone (7)
γ-lactone (8)
γ-lactone (9)
(b) optical
(c) TIC
(d) Ca+
(e) lignin
(f) m/z 279
(g) m/z 281
(h) m/z 297
(i) m/z 299
(j) m/z 321
(k) m/z 323
100 µm
max
min
(l)
(m)
(n)
(o)
Total Counts (x10⁴)
Total Counts (x10³)
261
263
(6)
(7)
(8)
(9)
K+
Na+
C3H7+
C4H7+
C5H7+
C5H9+
C6H9+
C7H11+
C7H13+
C7H15+
C8H13+
C8H11+
C9H11+
C9H13+
C10H13+
C10H15+
C16H23+
C16H25+
-[C2H2O]
-[H2C=C=C=CH2]
-CH2
-CO
-H2O
-[C2H4O2]
279
281
321
323
25x
10x
50x
5x

Figure S30: Distribution of γ-lactones 2-3, γ-lactone 4 rubrynolide, γ-lactone 5 rubrenolide, in sapwood (SW), transition zone (TZ) and heartwood (HW), respectively. (a)-(c) γ-lactone **4** **r**ubrynolide. (d)-(f) γ-lactone **5** rubrenolide. (g)-(i) γ-lactone **2**. (j)-(l) γ-lactone **3**.

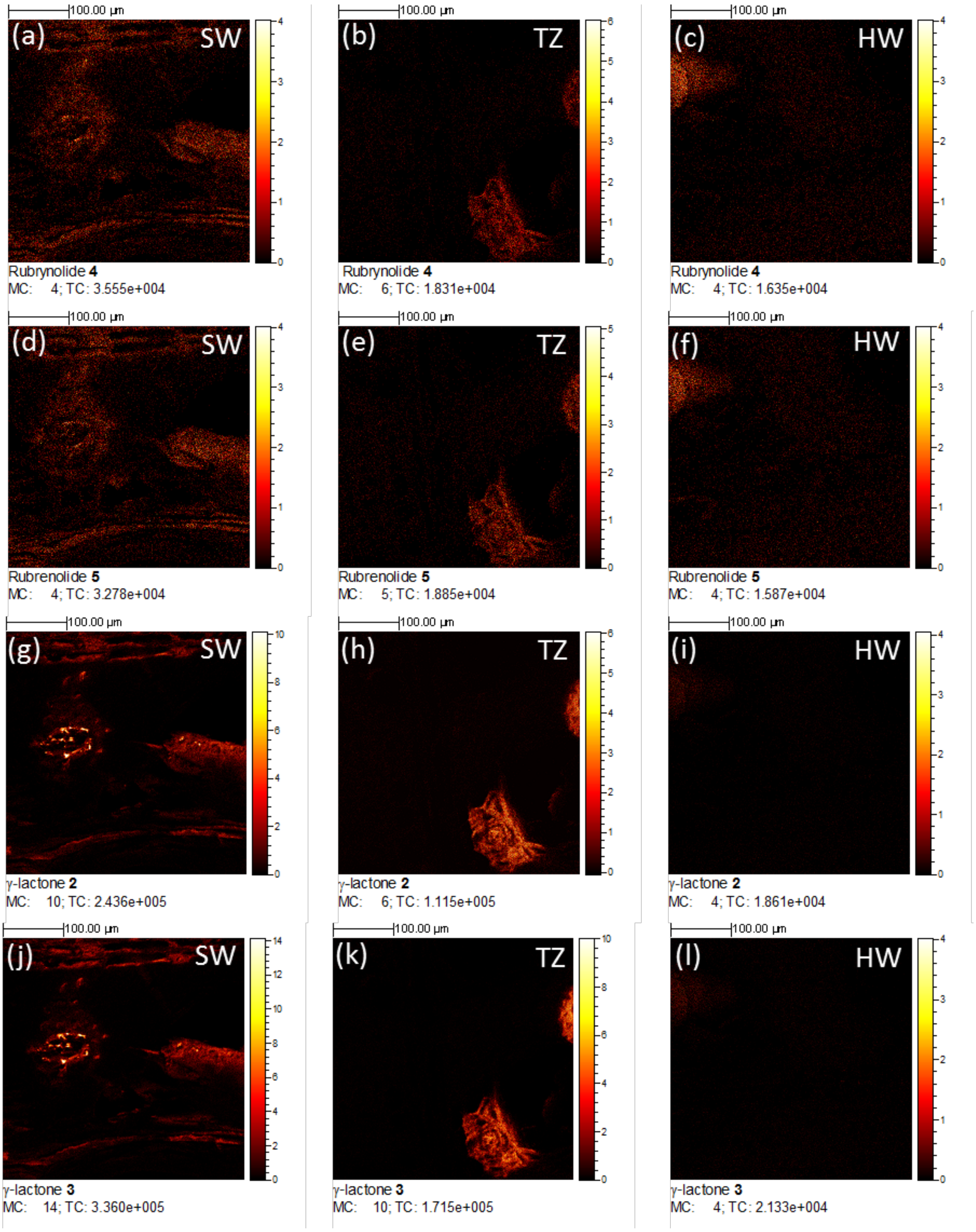

Figure S31: Total ion images of the analytical area at different depth during the argon cluster sputtering. Images are extracted from the 3D stack image displayed in Figure 5.

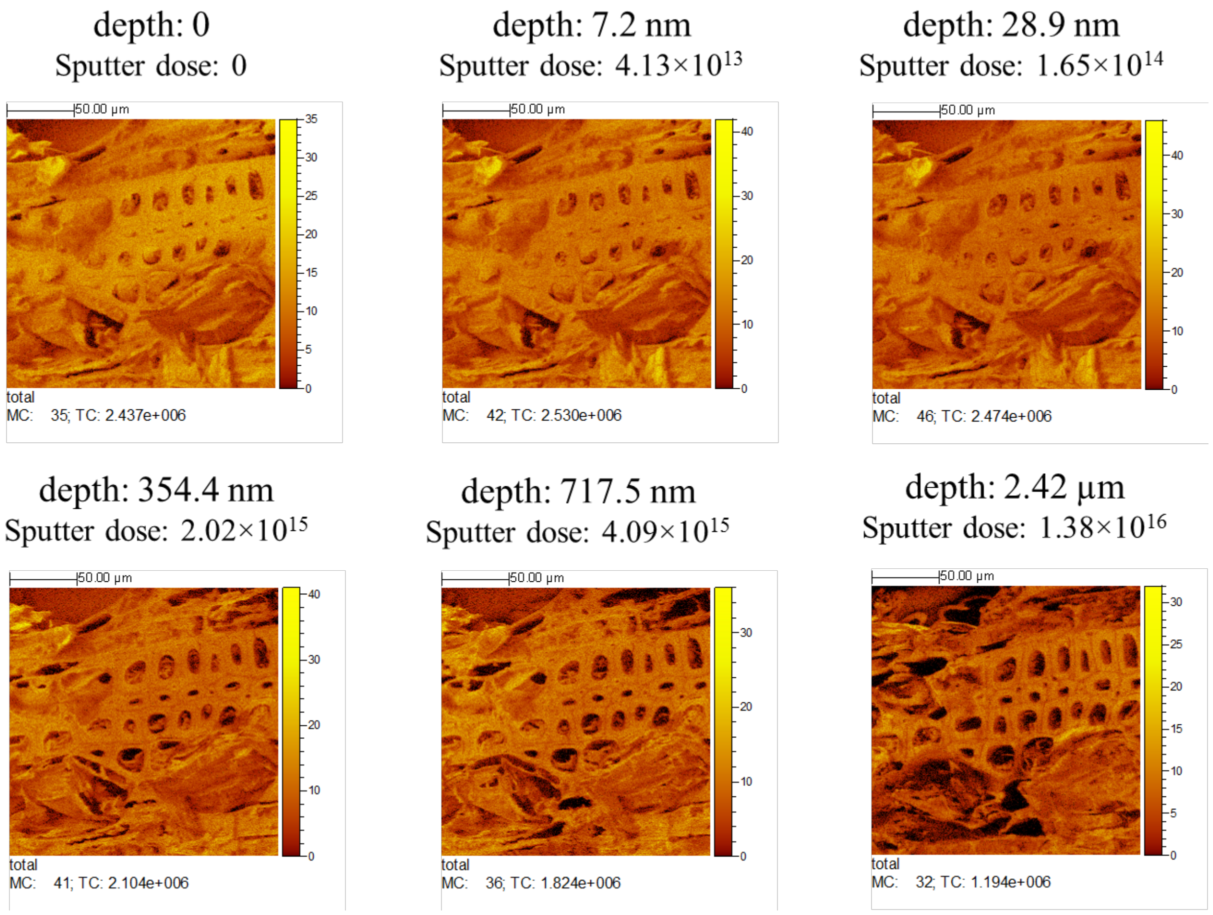

Figure S32: Argon cluster sputter depth measurement. (a) Optical image of a 20 µm thick *Sextonia rubra* wood section fixed on double side conductive tape. A: Analytical area. S: Sputter area. (b) Depth profile of the wood section fixed on the tape.

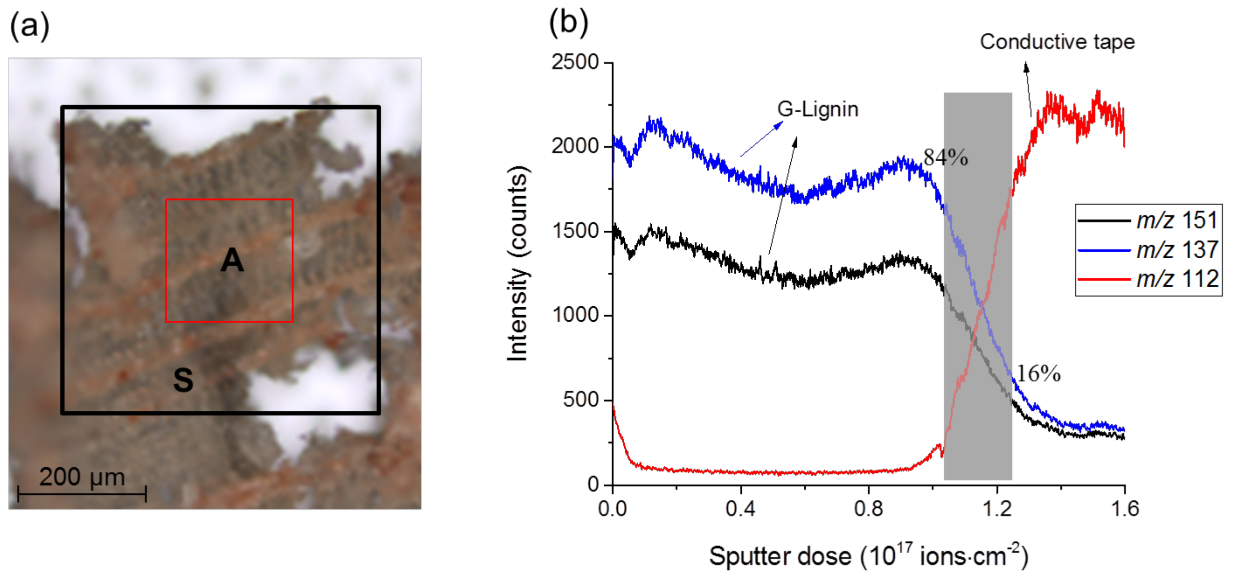

Supplement: Supplementary file 1 — SI revised [file 41598_2018_37577_MOESM1_ESM.pdf]
